# Naringin from Ganshuang granule inhibits inflammatory to relieve liver fibrosis through TGF-β-Smad signaling pathway

**DOI:** 10.1371/journal.pone.0304185

**Published:** 2024-06-10

**Authors:** Fuchun Wang, Jian Gan, Rui Li, Rui Yang, Xiaorong Mao, Shuang Liu, Yu Chen, Zhongping Duan, Junfeng Li

**Affiliations:** 1 Beijing Youan Hospital, Capital Medical University, Beijing, China; 2 Department of Gastroenterology, Yantai Affiliated Hospital of Binzhou Medical University, Yantai, Shandong, China; 3 Department of Obstetrics and Gynecology, Baiyin Pingchuan District People’s Hospital, Baiyin, Gansu, China; 4 Department of Infectious Disease, The First Hospital of Lanzhou University, Lanzhou, Gansu, China; 5 Infectious Disease Research Laboratory, The First Hospital of Lanzhou University, Lanzhou, Gansu, China; Harvard Medical School, UNITED STATES

## Abstract

**Objective:**

The present study aims to investigate the specific protective effects and underlying mechanisms of Ganshuang granule (GSG) on dimethylnitrosamine (DMN)-induced hepatic fibrosis in rat models.

**Methods:**

Hepatic fibrosis was experimentally evoked in rats by DMN administration, and varying dosages of GSG were employed as an intervention. Hepatocellular damage was assessed by measuring serum levels of aminotransferase and bilirubin, accompanied by histopathological examinations of hepatic tissue. The hepatic concentrations of platelet-derived growth factor (PDGF) and transforming growth factor-β1 (TGF-β1) were quantitated via enzyme-linked immunosorbent assay (ELISA). The expression of α-smooth muscle actin (α-SMA) within hepatic tissue was evaluated using immunohistochemical techniques. The levels of hepatic interferon-γ (IFN-γ), tumor necrosis factor-α (TNF-α), and a spectrum of interleukins (IL-2, IL-4, IL-6, IL-10) were quantified by quantitative real-time PCR (qRT-PCR). Additionally, hepatic stellate cells (HSCs) were cultured *in vitro* and exposed to TNF-α in the presence of naringin, a principal component of GSG. The gene expression levels of tissue inhibitor of metalloproteinase-1 (TIMP-1) and matrix metallopeptidase-1 (MMP-1) in these cells were also quantified by qRT-PCR. Proliferative activity of HSCs was evaluated by the Cell Counting Kit-8 assay. Finally, alterations in Smad protein expression were analyzed through Western blotting.

**Results:**

Administration of GSG in rats with fibrosis resulted in reduced levels of serum aminotransferases and bilirubin, along with alleviation of histopathological liver injury. Furthermore, the fibrosis rats treated with GSG exhibited significant downregulation of hepatic TGF-β1, PDGF, and TNF-α levels. Additionally, GSG treatment led to increased mRNA levels of IFN-γ, IL-2, and IL-4, as well as decreased expression of α-SMA in the liver. Furthermore, treatment with naringin, a pivotal extract of GSG, resulted in elevated expression of MMP-1 and decreased levels of TIMP-1 in TNF-α-stimulated HSCs when compared to the control group. Additionally, naringin administration led to a reduction in Smad expression within the HSCs.

**Conclusion:**

GSG has the potential to mitigate fibrosis induced by DMN in rat models through the regulation of inflammatory and fibrosis factors. Notably, naringin, the primary extract of GSG, may exert a pivotal role in modulating the TGF-β-Smad signaling pathway.

## Introduction

Liver fibrosis, which often leads to severe conditions such as liver cirrhosis and liver cancer, is a significant consequence of acute and chronic liver injuries [1]. The urgent demand for therapeutic interventions to reverse liver fibrosis persists. While effective clinical treatments for liver fibrosis are still limited, studies conducted on rodent models have demonstrated the possibility of preventing or even reversing the progression of liver fibrosis [2]. Additionally, clinical investigations have reported cases where liver fibrosis, and even cirrhosis, have shown regression [3]. The integration and synergy of traditional Chinese medicine and Western medicine offer promising prospects for the development of novel treatment approaches targeting liver fibrosis [4]. A previous study has provided evidence supporting the hepatoprotective effects of Ganshuang granule (GSG) [5]. However, the precise underlying mechanism is yet to be elucidated.

Transforming growth factor-β (TGF-β) plays a pivotal role as a pro-fibrotic cytokine across various phases of liver disease progression, encompassing the initial hepatic injury through to fibrosis, subsequently advancing to cirrhosis, and ultimately culminating in liver cancer [6]. During liver fibrosis evolution, TGF-β predominantly orchestrates the activation of quiescent hepatic stellate cells (HSCs), precipitating their transdifferentiation into the myofibroblast phenotype. This phenotype is a principal contributor to the accrual of extracellular matrix proteins (ECM) and acts as a key facilitator of fibrogenesis. The homeostasis of the ECM is delicately maintained by the interplay between matrix metalloproteinases (MMPs) and tissue inhibitors of metalloproteinases (TIMPs). Disruption of this equilibrium, characterized by an elevation in TIMPs and a reduction in MMPs, leads to impaired matrix degradation, a hallmark of liver fibrosis [7].

Our prior research has established that naringin, a key component of GSG, attenuates the activation of HSCs through the inhibition of the mammalian target of rapamycin (mTOR) pathway, thereby exerting an antifibrotic effect [8]. Furthermore, we have elucidated that GSG enhances the amelioration of cirrhosis and improve hepatic function, potentially through the modulation of T regulatory (Treg) cell differentiation—a process that may be influenced by HSCs—in a murine model of cirrhosis induced by carbon tetrachloride (CCl4) [9]. Nonetheless, the therapeutic impact of GSG in DMN-induced liver fibrosis remains to be elucidated. Consequently, this study is dedicated to investigating the mechanisms through which GSG and its pivotal constituent, naringin, mitigate liver damage prompted by DMN, employing both *in vivo* and *in vitro* models.

## Materials and methods

### 1. Ethics statement

This study was approved and supported by the Lanzhou University Institutional Animal Care and Use Committee and Scientific Program prior to its implementation (ethical application reference: LDYYLL2018–100).

### 2. Animals

Specific pathogen-free (SPF) male SD rats involved in the study were provided by the Lanzhou University Animal Laboratory.

### 3. Reagents, and antibodies, drugs

Dimethylnitrosamine (Macklin Inc., Shanghai); bicyclol (Union Pharmaceutical Factory, Beijing); TGF-β1 enzyme-linked immunosorbent assay(ELISA) kit and platelet-derived growth factor-BB (PDGF-BB) ELISA kit (Elabscience Biotechnology Co., Ltd., Wuhan); The plate reader of ELISA(Varioskan Flash, Thermo Fisher Company, USA)UV spectrophotometry, Prestained protein marker and ECL-PLUS/Kit (Thermo Fisher Nano Drop, USA); ECL western blotting kit (Thermo Fisher Scientific, USA); enzyme labeling instrument (Thermo Scientific Varioskan Flash, Massachusetts, USA); Streptavidin Biotin-peroxidase Complex (SABC) Kit, DAB (Boster Biological Technology., Ltd., Wuhan); SDS-polyacrylamide gel electrophoresis (PAGE, VE-180, Tanon Science & Technology Co., Ltd., Shanghai); Citric acid buffer (pH 6.0) (Solebo Technology Co., Ltd., Beijing); RNA extraction kit, reverse transcription kit and fluorescent quantitative PCR kit (QIAGEN, Germany); rat GAPDH, TIMP-1, MMP-1, TNF-α, IFN-γ, IL-2, TGF-β (Takara Biomedical Technology, Japan); GAPDH antibody (Beyotime Biotechnology, AF1186, Shanghai); anti-α-SMA antibody (Cell Signaling Technology Inc., 19245, USA).

GSG was produced by Baoding Tianhao Pharmaceuticals Co., Ltd., with the approval number being Guoyao Zhunzi Z20027671. The primary constituents of GSG include: Radix Bupleuri (Chaihu), Paeoniae Radix Alba (Baishao), Angelicae Sinensis Radix (Danggui), Poria Cocos (Schw.) Wolf. (Fuling), Atractylodes Macrocephala Koidz. (Baizhu), Aurantii Fructus (Zhike), Codonopsis Radix (Dangshen), Carapax Trionycis (Biejia), Taraxacum mongolicum Hand.-Mazz. (Pugongying), Polygoni Cuspidati Rhizoma Et Radix (Huzhang), Prunellae Spica (Xiakucao), Radix Salviae (Danshen), Persicae Semen (Taoren), among others.

### 4. Experimental procedures

Thirty Sprague-Dawley (SD) rats were evenly and randomly allocated into six groups, with five rats per group: five model groups and one blank control group. The model groups were administered 0.5% DMN [10] at a dosage of 2 mL/kg body weight via intragastric gavage thrice weekly for a duration of two weeks. The blank control group was treated with an equivalent volume of normal saline in lieu of DMN. Following the cessation of DMN administration, the rats were subjected to an additional week of treatment as follows: (1) The blank control group continued to receive standard chow; (2) The model group, in addition to regular feed, received distilled water via gavage, matching the volume of GSG treatment; (3) The low-dose GSG group was administered 81 mg/day of GSG via gavage alongside their normal diet; (4) The medium-dose GSG group received 162 mg/day of GSG via gavage in addition to their usual diet; (5) The high-dose GSG group was given 324 mg/day of GSG via gavage, also with their regular diet; (6) The positive control group was treated with 324 mg/day of bicyclol via gavage, complemented by standard feed. At the termination of the experiment, euthanasia was induced with an intraperitoneal injection of 1% pentobarbital sodium at a dose of 80 mg/kg, followed by exsanguination via aortic blood withdrawal. The collected blood was obtained through the abdominal aorta and subjected to centrifugation at 3500 rpm for 20 minutes.

### 5. Liver function analyses

Serological analyses were performed to ascertain the concentrations of alanine aminotransferase (ALT), aspartate aminotransferase (AST), and total bilirubin (TBIL), which serve as indicators of hepatocellular damage. These parameters were measured using the Olympus AU400 series automated biochemical analyzer, manufactured in Japan.

### 6. Pathological evaluation

Liver tissue specimens were meticulously cleansed with chilled saline to eliminate any residual blood. Subsequently, the samples were fixed in a 4% paraformaldehyde solution for a period of 48 hours, embedded in paraffin, and sectioned to a thickness of 4 μm using a Leica RM2235 microtome. Hematoxylin and eosin (HE) staining was performed, adhering to the protocol established in prior studies conducted by our research team [11]. An experienced pathologist, Dr. Tian, conducted a thorough microscopic examination of the stained sections utilizing a Nikon ECLIPSE 80i microscope and compiled the findings into a detailed report.

### 7. Cell experiments

The T6 lineage of HSCs, maintained at the Artificial Liver Center, Beijing YouAn Hospital, Capital Medical University, Beijing, China, is a well-established cell line. For experimental purposes, sub-cultures of HSCs demonstrating robust growth and proliferation were selected. These cells were propagated in RPMI-1640 medium supplemented with 10% fetal bovine serum under controlled environmental conditions of 37°C, 5% CO2, and 95% relative humidity within a cell culture incubator. The medium was replenished every other day until the cells attained 80–90% confluence, at which point they were detached using 0.05% trypsin. For experimental interventions, cells were allocated into three distinct groups: (1) a control group consisting of untreated HSCs; (2) a TNF-α group, wherein cells were exposed to 10 ng/mL TNF-α to simulate the HSCs activation induced by DMN; and (3) a naringin intervention group [8], where cells received a 30-minute pretreatment with 10 ng/mL TNF-α followed by the administration of 20 ng/L naringin, as based on prior research. Post 48-hour incubation, cell supernatants were harvested via centrifugation for MMP-1 and TIMP-1 expression analysis. Additionally, HSCs viability was quantified using the Cell Counting Kit-8 (CCK-8) in conformity with the manufacturer’s protocol.

### 8. ELISA assay

The 10% tissue homogenate was prepared in accordance with the double antibody sandwich ELISA protocol to determine the concentrations of TGF-β1, PDGF-BB, and TNF-α using the corresponding ELISA kits, following the manufacturer’s instructions. Each well received 100 μL of either the standard substance or the sample, which was then incubated at 37°C for 90 min. Subsequently, the liquid was removed from the wells. Next, 100 μL of the biotinylated antibody working solution (TGF-β1/PDGF-BB/TNF-α) was added and incubated at 37°C for 60 min. After three washes, 100 μL of the enzyme conjugate working solution was added and incubated at 37°C for 30 min. The wells were washed five times and then 90 μL of the substrate solution (TMB) was added, followed by incubation at 37°C for 15 min. Finally, 50 μL of the termination solution was added, and the optical density (OD) value at a wavelength of 450 nm was immediately measured. The concentrations of TGF-β1, PDGF-BB, and TNF-α in the samples were calculated using the standard curve.

### 9. Immunohistochemistry

The expression of α-smooth muscle actin (α-SMA) in liver tissue was detected using the SABC method, following the instructions provided with the SABC Kit. Paraffin sections were dewaxed and hydrated, and endogenous catalase was removed using 3% H2O2. Rabbit anti-rat α-SMA monoclonal antibody (1:50) was applied as the primary antibody and incubated overnight at 4°C, while PBS served as the blank control. The sections were stained with DAB, washed with distilled water, counterstained with hematoxylin, and rinsed again with distilled water. Subsequently, the sections were rapidly dehydrated using 75%, 85%, 95% ethanol, and anhydrous ethanol, followed by clarification with xylene. Finally, the samples were mounted on a resin mount. Under a light microscope, positive staining was observed as a granular deposition of brown-yellow in the designated areas.

### 10. Quantitative polymerase chain reaction (qPCR)

Total ribonucleic acid (RNA) was extracted using an RNA extraction kit, following the manufacturer’s instructions. The extracted RNA was then subjected to centrifugation and UV spectrophotometry, and the resulting pellet was stored at –20°C. Subsequently, the RNA was reverse transcribed into complementary DNA (cDNA) using a reverse transcription kit. The mRNA levels of TNF-α, IFN-γ, IL-2, IL-4, IL-6, IL-10, MMP-1, and TIMP-1 were determined by qPCR using the SYBR Green PCR Kit on a real-time PCR system (Roche Light Cycler 480II, Switzerland). The housekeeping gene GAPDH was selected as the internal reference, and the specific primer sequences are provided in Table 1. The PCR reaction system consisted of 20 μL, and the reaction conditions included pre-denaturation at 94°C for 2 min, followed by 40 cycles of denaturation at 94°C for 5 s, annealing at 60°C for 15 s. The relative quantitative threshold cycle (Ct) value of each primer was normalized to the internal primer. The ΔCt represented the difference between the Ct value of the target gene and the internal gene. Finally, the 2-ΔCt values were obtained and compared between groups.

**Table 1:** Primer sequences for qPCR analysis.

Gene	Primer sequences (5’→3’)
Rat TNF-α	Forward: ATGGGCTCCCTCTCATCAGT
	Reverse: GCTTGGTGGTTTGCTACGAC
Rat IFN-γ	Positive: ACCCACAGATCCAGCACAAAGC
	Reverse: CCAGAATCAGCACCGACTCCTT
Rat IL-2	Positive: CACTGACGCTTGTCCTCCTT
	Reverse: GTTTCAATTCTGTGGCCTGCT
Rat IL-4	Positive: CGTGATGTACCTCCGTGCTTGA
	Reverse: TCAGTGTTGTGAGCGTGGACTC
Rat IL-6	Positive: GCCCACCAGGAACGAAAGTCAA
	Reverse: AGGCAACTGGCTGGAAGTCTCT
Rat IL-10	Positive: CCTGCTCTTACTGGCTGGAGTG
	Reverse: TGGGTCTGGCTGACTGGGAA
MMP-1	Positive: CGCCTTCTACAGAGGAGACCAT
	Reverse: GGTGGGAATGTGTGAGCAAGTC
TIMP-1	Positive: CACGCTAGAGCAGATACCACG
	Reverse: GTAGGCGAACCGGAAACCTG
GAPDH (internal reference)	Positive: CAGTGCCAGCCTCGTCTCAT
	Reverse: CAGCCTTGACTGTGCCGTTG

(Note. TNF-α: tumor necrosis factor-α; IFN-γ: Interferon γ; IL-2: interleukin-2; IL-4: interleukin-4; IL-6: interleukin-6; IL-10: interleukin-10; MMP-1: matrix metalloproteinases-1; TIMP-1: tissue inhibitors of metalloproteinases-1; GAPDH: glyceraldehyde phosphate dehydrogenase)

### 11. Western blotting analysis

Liver tissues were lysed using RIPA buffer in an ice bath, with a ratio of 150–250 μL of lysate per 20 mg of tissue. After centrifugation, the protein concentration in the supernatant was determined using the BCA method, and the concentration of each sample was adjusted to 2 μg/μL. The exact amount of total proteins was separated using 12% acrylamide gel electrophoresis and transferred to PVDF membranes at 4°C for 150 min under a constant current of 300 mA. The PVDF membranes (IPVH00010, Millipore) were blocked for 1 h at room temperature and then incubated overnight with the primary antibody (diluted in a 1:1000 blocking solution) at 4°C. After washing the membranes, the corresponding secondary antibody, diluted in the blocking solution, was applied and incubated at room temperature for 1.5 h. The membranes were then washed four times with TBST. Finally, the protein bands on the membrane were visualized using an ECL Western blotting kit. Densitometry analysis was performed using the ImageJ 2x software (Rawak Software Inc., Stuttgart, Germany).

### 12. Cell proliferation trial of CCK-8

A total of 100 μL of cell suspension was seeded in each well of a 96-well plate and pre-cultured in an incubator (37°C, 5% CO2) for 24 h. Subsequently, 10 μL of the substance at various concentrations was added to the culture plate, including HSCs cell suspension, HSCs cell suspension with TNF, and HSCs cell suspension with TNF followed by naringin. The culture plate was then incubated in the incubator for an appropriate period (24 h, 48 h, 72 h). After that, 10 μL of CCK-8 solution was added to each well, and the culture plate was further incubated for 1–4 h. Finally, the absorbance at 450 nm was measured using an enzyme labeling instrument.

### 13. Statistical analyses

Data analysis was performed using GraphPad Prism 7 software (GraphPad Software Inc., San Diego, CA, USA). All data analyzed in this study were continuous variables. To compare the differences in variables between two groups, an independent sample t-test was utilized if both groups’ variables conformed to a normal distribution. If one group’s variable did not satisfy the normal distribution, the Mann-Whitney U test was employed. For the comparison of differences in variables among multiple groups, a one-way analysis of variance (ANOVA) was applied if all variables adhered to a normal distribution. If one group’s variables did not meet the normal distribution criteria, the Kruskal-Wallis test was conducted. *P*-value < 0.05 was considered statistically significant.

## Results

### 1. GSG mitigates liver injury and fibrosis in a rat model

**Fig 1:**
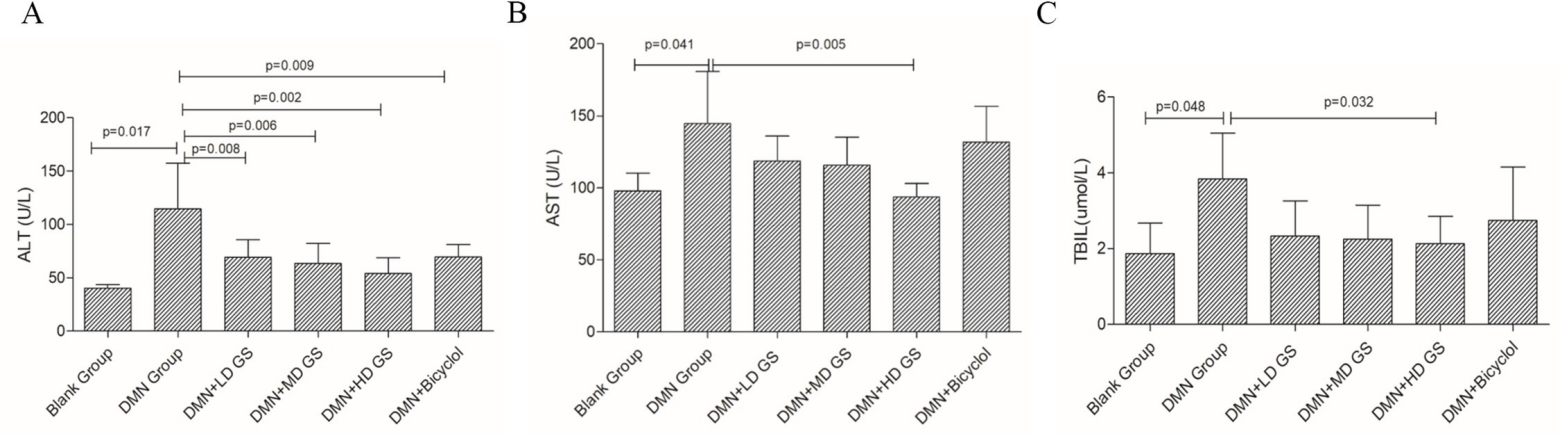
Serum ALT, AST, and TBIL levels of rats in each group. (Note. Blank group: nothing; DMN group: only DMN; LD: low dose of GSG; MD: a middle dose of GSG; HD: high dose of GSG).

**Fig 2:**
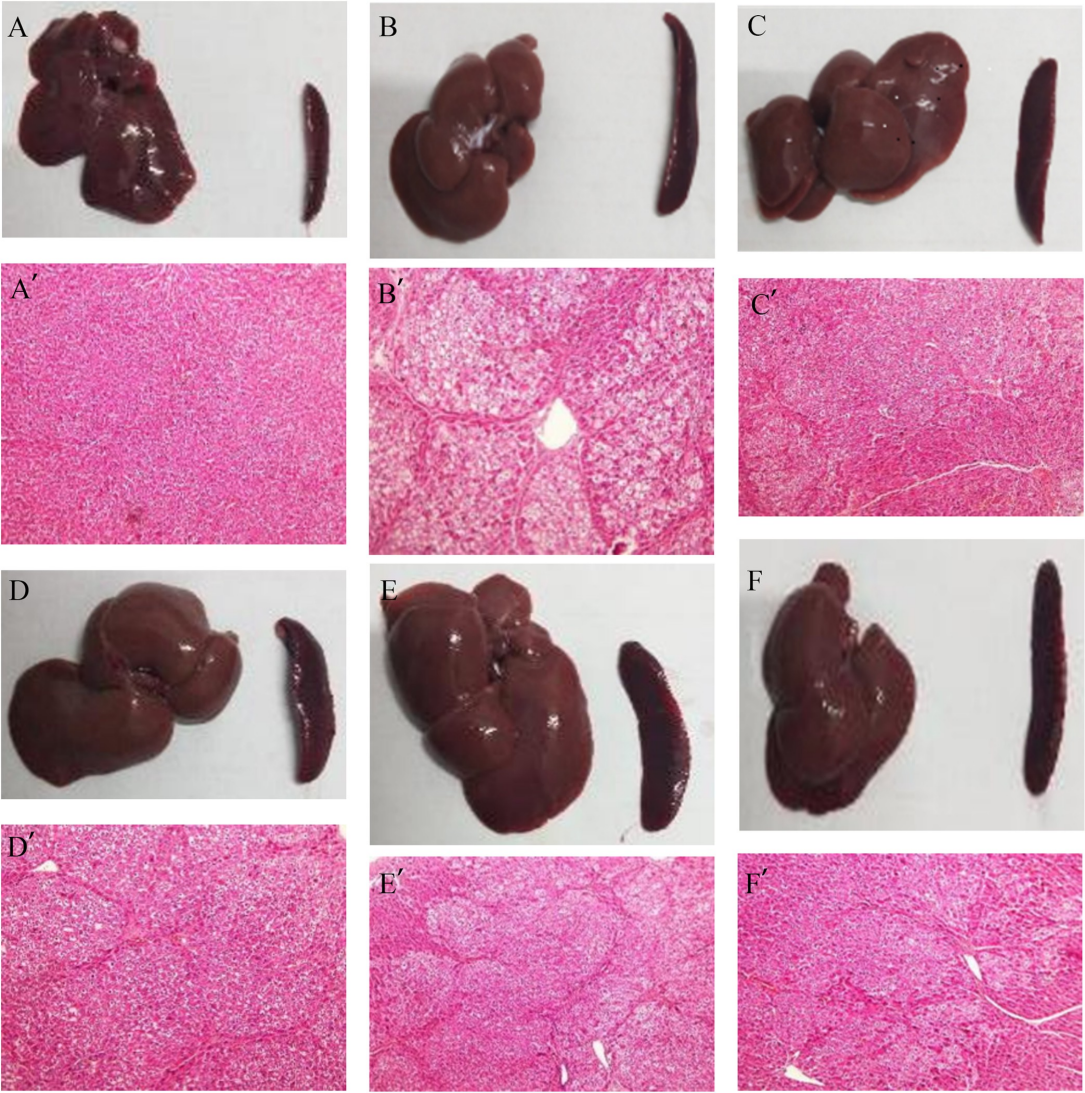
Morphological alterations of the liver and spleen, alongside the pathological features at 4× magnification, were evaluated post DMN exposure under varied intervention protocols. (Note. A& A’: blank control group; B& B’: model group; C& C’: low-dose GSG group; D& D’: middle-dose GSG group; E& E’: high-dose GSG group; F& F’: bicyclol group).

The hematological indices revealed that, in comparison with the model group, both the GSG treatment and the positive control medication significantly decreased ALT levels (*P* < 0.05), with the high-dose GSG group demonstrating a notable reduction (*P* = 0.002) (Fig 1A). Concerning AST and TBIL levels, only the high-dose GSG treatment was effective in reducing their concentrations (*P* < 0.05) (Fig 1B and 1C). Additionally, macroscopic examination of liver and spleen specimens from each experimental group indicated that the liver surface in the model group was notably irregular, which showed mild improvement in the intervention groups, along with an increase in spleen size (Fig 2A–2F). Moreover, HE staining corroborated the destruction of the normal hepatic lobular architecture, the formation of pseudolobules, and the onset of early cirrhosis in the model group—findings that align with previous studies demonstrating DMN-induced hepatic fibrosis. The livers of rats treated with GSG and the positive control, bicyclol, exhibited fewer fibrous connective tissues and pseudolobules in comparison to those in the model group (Fig 2A’–2F’). These results suggest that the extent of glycofibrosis was significantly more pronounced in the model group than in the GSG-treated group, indicating that GSG exerts an anti-fibrotic effect in the DMN-induced hepatic fibrosis rat model. Moreover, the high-dose GSG group displayed a significantly superior improvement over the positive control group.

### 2. In DMN-induced rat models, GSG modulates the expression of various cytokines

**Fig 3:**
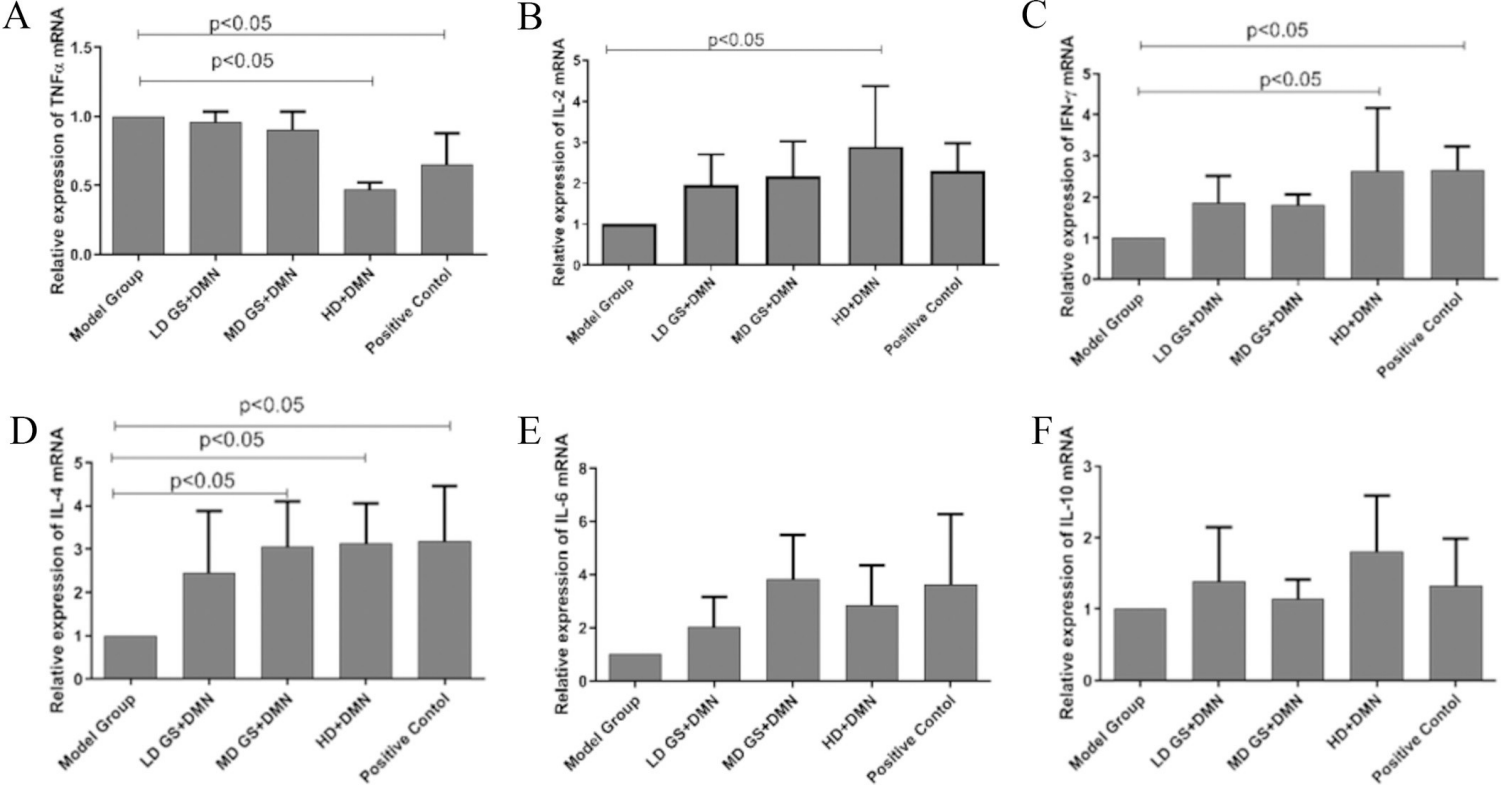
Effects of GSG on the expression of key genes in DMN-induced liver injury.

DMN-induced hepatic injury offers a solid foundation for the development of an immune-mediated liver injury model. To elucidate the precise impact of GSG on DMN-induced hepatic injury, cytokine concentrations were measured in rat serum. Rats administered with DMN exhibited decreased levels of interleukins (IL-2, IL-4, IL-6, IL-10), IFN-γ, and elevated levels of TNF-α compared to those in the GSG-treated and positive control groups (Fig 3A–3F). As indicated in these figures, the low- and middle-dose GSG treatment groups, as well as the positive control group receiving bicyclol, showed a significant reduction in TNF-α expression in the DMN-induced liver injury model (*P* < 0.05), with the decrease being most pronounced in the high-dose GSG group (Fig 3A). Furthermore, GSG treatment enhanced the expression of innate immune mediators such as IL-2, IL-4, and IL-10 (Fig 3B, 3D and 3F), as well as anti-fibrotic cytokines like IFN-γ (Fig 3C), particularly notable in the high-dose GSG group (*P* < 0.05). These findings suggest a potential immunomodulatory and anti-fibrotic role of GSG in the context of chemically induced liver injury.

### 3. GSG suppresses activation of HSCs in rat models

**Fig 4:**
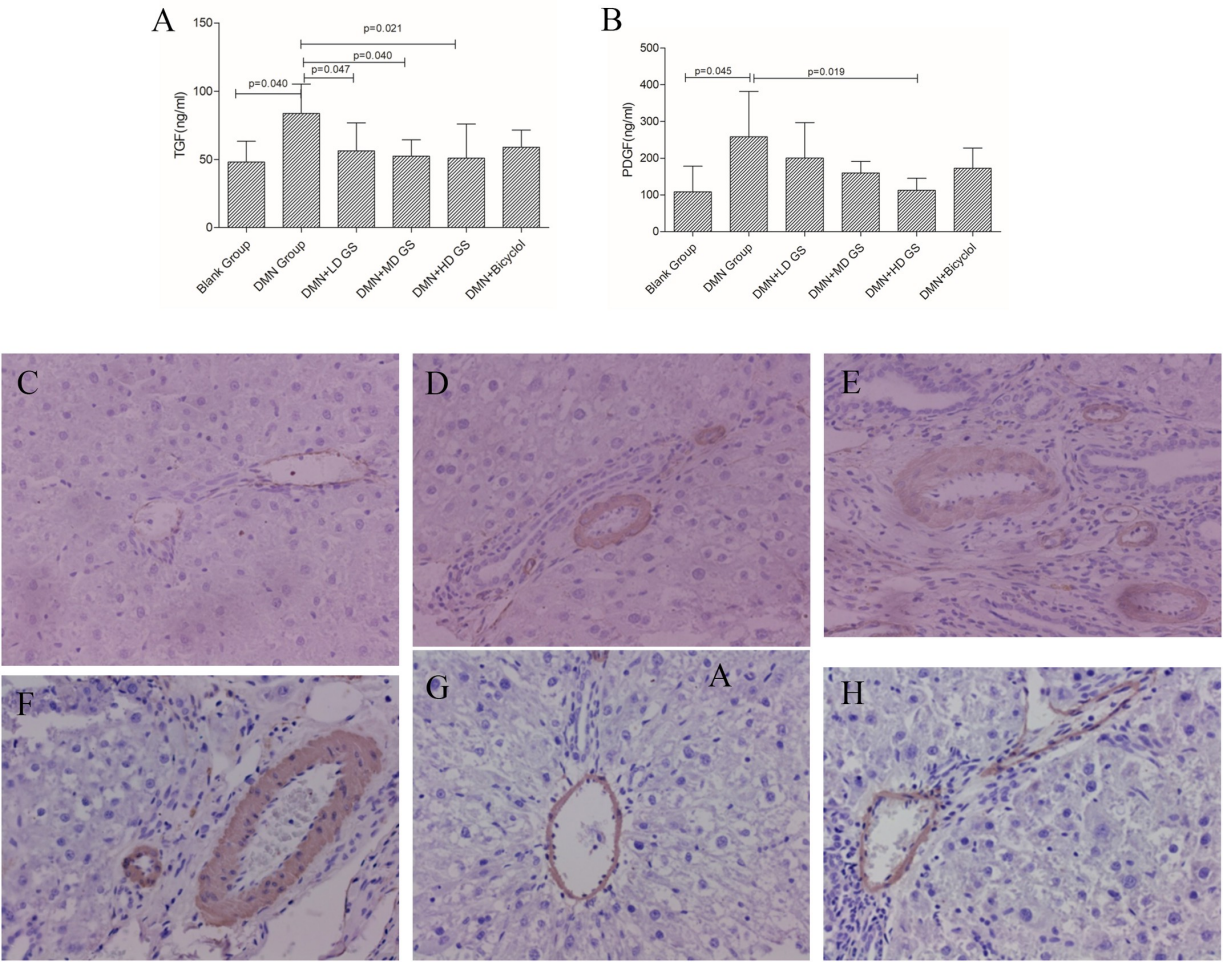
Effects of GSG on the key factors and HSCs activation of DMN-induced liver injury. (Note. C: blank control group; D: model group; E: low-dose GSG group; F: middle-dose GSG group; G: high-dose GSG group; H: bicyclol group).

ELISA outcomes indicated differential concentrations of TGF-β1 and PDGF-BB, pivotal regulators of HSCs activation, across the study groups. Relative to the model group, TGF-β1 levels were significantly diminished in the GSG-administered group (*P* < 0.05), with the most substantial decrease observed in the high-dose GSG cohort (*P* = 0.021) (Fig 4A). Conversely, PDGF-BB expression was markedly attenuated in the high-dose GSG group only (*P* < 0.05) (Fig 4B). These results indicate that GSG may exert anti-fibrotic properties via the suppression of HSCs activation. Subsequently, the expression of α-SMA, an established marker of activated HSCs [12, 13], was assessed through immunohistochemical analysis to further investigate the effect of GSG on DMN-induced hepatic fibrosis in rats. Compared with the blank control group (Fig 4C), α-SMA levels were significantly elevated in the DMN group (Fig 4D). However, α-SMA expression was notably diminished in the livers of rats treated with GSG (Fig 4E–4G) or bicyclol (Fig 4H). These findings suggest that GSG mitigates DMN-induced liver fibrosis in rats potentially by inhibiting the activation of HSCs, thereby reducing α-SMA production.

### 4. Naringin diminishes activation of HSCs *in vitro*

**Fig 5:**
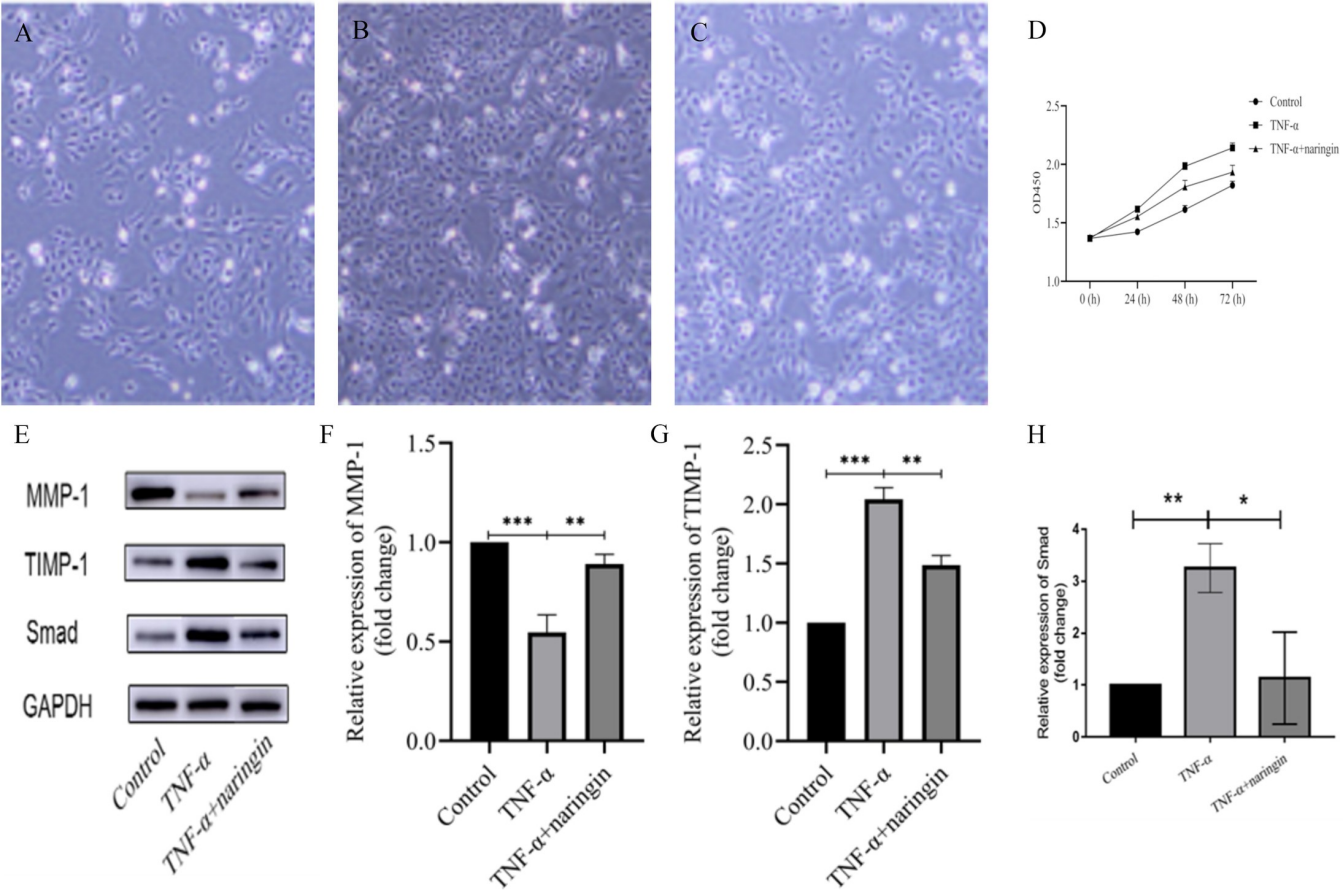
Naringin may affect the TGF-β/Smad signaling pathway to regulate the activity of HSCs. (Note. A: The blank control group of HSCs under an electron microscope. B: Increased number of HSCs after TNF intervention. C: The number of HSCs decreased after the addition of naringin. D: Changes in the number of HSCs in each group. E: MMP-1, TIMP-1, and Smad proteins were determined by the WB method. F: The relative expression level of MMP-1 mRNA measured by the qRT-PCR method. G: The relative expression level of TIMP-1 mRNA measured by the qRT-PCR method. H: The relative expression level of Smad mRNA measured by the qRT-PCR method. *: *P* <0.05, **: *P* <0.01, ***: *P* <0.001).

Previous studies have highlighted that hepatic fibrosis development is contingent upon a disruption in the equilibrium of matrix metalloproteinases and their inhibitors—specifically, a diminution in MMP-1 and an augmentation in TIMP-1 expression [14, 15]. In the present investigation, TNF-α was employed to incite HSCs, thereby fabricating an in vitro liver fibrosis model. The concentration of MMP-1 and TIMP-1 was quantified to assess the efficacy of naringin in attenuating the activation of HSCs. Following the administration of TNF-α, a considerable decrement in MMP-1 levels was observed in comparison to the blank control group (*P* < 0.001) (Fig 5F), concomitant with an increment in TIMP-1 expression (*P* < 0.001) (Fig 5G). Contrarily, subsequent to the TNF-α induction, treatment with naringin elicited a significant upsurge in MMP-1 (*P* < 0.01) (Fig 5F) and a decrease in TIMP-1 levels (*P* < 0.01) (Fig 5G). Furthermore, electron microscopy images at 4× magnification distinctly exhibited a substantial variance in the quantity of HSCs post-addition of TNF-α followed by naringin intervention (Fig 5A–5C). Concurrently, the OD values escalated over time relative to the control group. Notably, the OD values surged markedly after the sequential addition of TNF-α and naringin, situating between the benchmarks set by the blank control group and the TNF-α group (Fig 5D). These findings infer that while TNF-α can provoke the activation and proliferation of HSCs, naringin may exert suppressive effects on the proliferation and activation of HSCs, delineating its potential therapeutic role in the modulation of liver fibrosis.

### 5. Naringin inhibits the activation of HSCs via the TGF-β-Smad signaling pathway

Given that TGF-β1 is a pivotal mediator of the Smad signaling pathway and is known to facilitate the progression of liver fibrosis [16, 17], the present study meticulously examined the specific role of the TGF-β-Smad axis in the context of naringin-mediated inhibition of HSCs activation. Induction with TNF-α was employed to stimulate the activation of HSCs, which correspondingly manifested in an increased expression of Smad proteins. Conversely, the addition of naringin resulted in a downregulation of Smad expression (Fig 5E and 5H). These observations point to a probable mechanism by which naringin deactivates HSCs, possibly through the impediment of the Smad signaling cascade, thereby contributing to its anti-fibrotic effects. TNF-α was used to promote the activation of HSCs; meanwhile, relative expression of Smad was observed to be increased. In contrast, when naringin was added, the expression of Smad was decreased (Fig 5E and 5H). These results may suggest that the inactivation of HSCs by naringin is likely due to the inhibition of the Smad pathway.

## Discussion

Our prior research has substantiated that naringin, the principal bioactive constituent of GSG, exerts an inhibitory effect on the mTOR, thereby dampening the activity of HSCs and facilitating an anti-fibrotic response [8]. Furthermore, we observed that GSG ameliorated cirrhosis and enhanced liver function by partially suppressing the differentiation of regulatory Tregs, a process potentially orchestrated by HSCs in a murine model of cirrhosis elicited by carbon tetrachloride (CCl4) [9]. This investigation marks the inaugural demonstration of GSG, particularly its naringin component, thwarting fibrosis by modulating the TGF/Smad pathway *in vitro*.

DMN is well-recognized for its hepatotoxicity in rats, manifesting fibrotic liver characteristics, prompting collagen accumulation, and precipitating hepatocyte apoptosis. Our findings revealed that rats with DMN-induced liver fibrosis presented elevated serum levels of ALT, AST, TBIL, and α-SMA, alongside severe pseudolobular formation upon histopathological examination. Post-administration of GSG, there was an observable attenuation of liver injury in the fibrotic animals, underscoring the therapeutic potential of GSG in the amelioration of liver fibrosis.

TGF-β1 is recognized as one of the most potent fibrogenic agents, instrumental in the activation of HSCs and playing a vital role in the cascade of hepatocellular damage and hepatic fibrosis [18]. The TGF-β1/Smad signaling pathway is significantly implicated in the advancement of hepatic fibrosis [19]. Additionally, during liver injury, activated HSCs secrete latent TGF-β, which further propagates fibrosis by establishing an autocrine positive feedback loop via Smad2/Smad3 activation [16, 17]. Furthermore, PDGF serves as a potent mitogen, fostering the proliferation of HSCs [20, 21], and is critically involved in the mitotic activities during HSCs activation and fibrosis progression [22]. In our current investigation, we observed a marked reduction in the levels of TGF-β1 and PDGF-BB in the liver tissues, along with significant histopathological amelioration in the fibrotic liver of rats treated with GSG. Notably, in the high-dose GSG group, subjected to DMN-induced liver injury, there was a substantial decrement in TGF-β1 and PDGF-BB levels, implying that GSG may attenuate the activation and proliferation of HSCs through the inhibition of the TGF-β signaling axis, thereby mitigating the severity of liver fibrosis.

The interplay between inflammatory and fibrotic cells is characterized by a self-propagating positive feedback loop that perpetuates the advancement of fibrosis [23]. IFN-γ is well-documented for its immunoregulatory and antifibrotic attributes, capable of attenuating ECM deposition through the inhibition of HSCs activation or by reverting activated HSCs to a quiescent state [24]. Consequently, mitigating intrahepatic inflammation stands as a viable strategy for decelerating fibrosis progression. Our findings indicate that GSG administration reduced the expression of the pro-inflammatory cytokine TNF-α, elevated the levels of the antifibrotic agent IFN-γ, and modulated innate immune mediators, including IL-2 and IL-4. These alterations suggest that the bioactive constituents of GSG may attenuate hepatic fibrosis and potentially revert the fibrotic process by curtailing inflammatory responses.

To elucidate the precise antifibrotic mechanism of GSG, an in vitro approach was adopted wherein HSCs were stimulated with TNF-α to replicate the cellular paradigm observed in DMN-induced HSCs [22, 23]. Naringin, the predominant active monomeric component of GSG [8], was employed in this in vitro investigation.

TIMP-1 acts as a specific antagonist to MMP-1, forming a covalent bond with MMP-1, thereby impeding its enzymatic function [25, 26]. MMP-1, a principal matrix-degrading enzyme, is implicated in the degradation of various extracellular matrix components. An imbalance in the expression levels of MMP-1 and TIMP-1 has been observed in fibrotic liver tissue, characterized by elevated TIMP-1 and reduced MMP-1 expression [14, 15]. Following TNF-α treatment, MMP-1 expression in HSCs was significantly reduced in comparison to the blank control. In contrast, TIMP-1 expression exhibited an increase. Naringin treatment, however, resulted in an upregulation of MMP-1 and a concurrent downregulation of TIMP-1 levels relative to the TNF-α treated group. Additionally, OD values of HSCs treated with both TNF-α and naringin significantly diminished compared with cells stimulated solely with TNF-α, indicating that naringin has the potential to inhibit TNF-α induced HSCs activation and proliferation. Furthermore, TNF-α stimulation alone resulted in an upsurge in Smad protein expression. Conversely, the co-administration of naringin with TNF-α led to a reduction in Smad expression in stimulated HSCs, suggesting that naringin may impede the Smad signaling pathway, thus contributing to HSCs inactivation. It is also indicated that the activation of the Smad pathway could negate the suppressive impact of naringin on HSCs activation.

Several limitations within this study warrant discussion. Firstly, while our results indicate that naringin may attenuate HSCs activation by modulating MMP-1 and TIMP-1 levels *in vitro*, it remains uncertain whether naringin exerts regulatory effects on multiple types of MMPs and TIMPs concurrently. Moreover, given the complexity of the GSG composition, the sole contribution of naringin, as well as potential interactions among various constituents, have yet to be fully elucidated. Addressing these considerations will form the cornerstone of our subsequent research endeavors, which are currently progressing.

In summary, the present study enhances the current comprehension of the anti-fibrotic mechanisms underlying GSG. Additionally, we have ascertained that naringin within GSG impedes HSCs activation by inhibiting the TGF-β-Smad signaling pathway and that GSG may promote apoptosis in activated HSCs via critical mediators such as TGF and PDGF, thereby effectively mitigating DMN-induced hepatic fibrosis in rats. Our findings furnish a theoretical framework for the potential use of GSG in the reversion of liver fibrosis and extend new prospects for the integrative treatment of liver fibrosis utilizing both Western and traditional Chinese medical practices.

## Supporting information

S1 File (ZIP)

S1 Raw data (ZIP)
