# Supplementary figures and images for: Naringin from Ganshuang granule inhibits inflammatory to relieve liver fibrosis through TGF-β-Smad signaling pathway
Source: PLoS One. 2024 Jun 10;19(6):e0304185. doi: 10.1371/journal.pone.0304185 (PMC11164354; doi:10.1371/journal.pone.0304185)

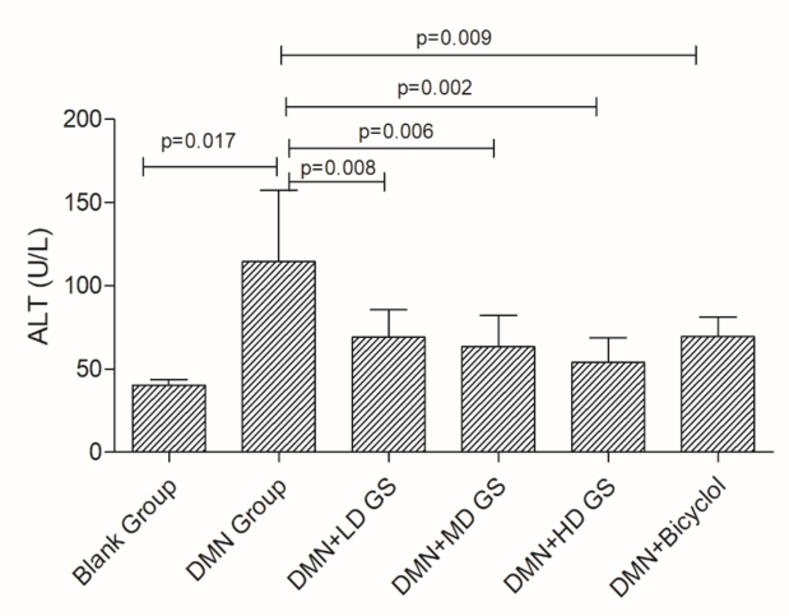

Supplement: S1 File — (ZIP) [file pone.0304185.s001.zip › Single Image-TIF-PACE/Fig1/Fig1A.tif]

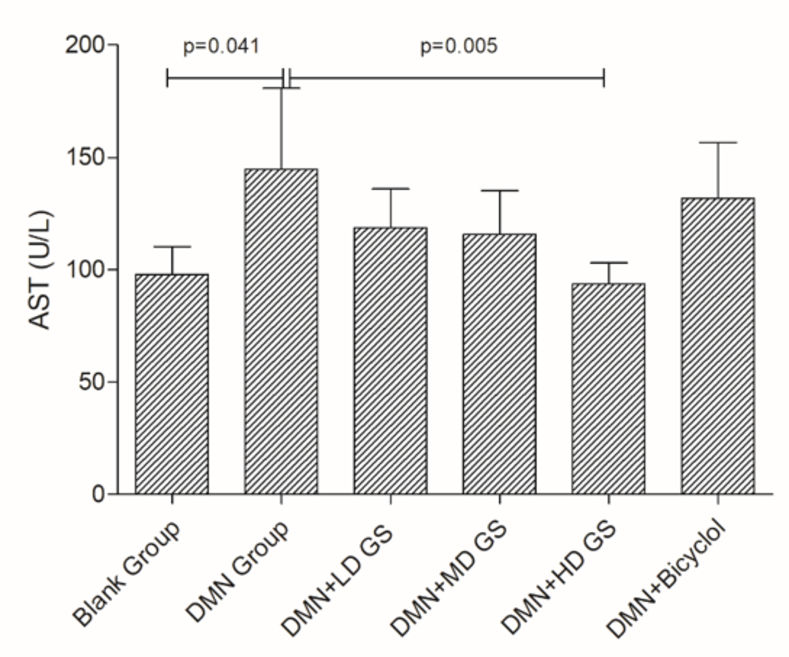

Supplement: S1 File — (ZIP) [file pone.0304185.s001.zip › Single Image-TIF-PACE/Fig1/Fig1B.tif]

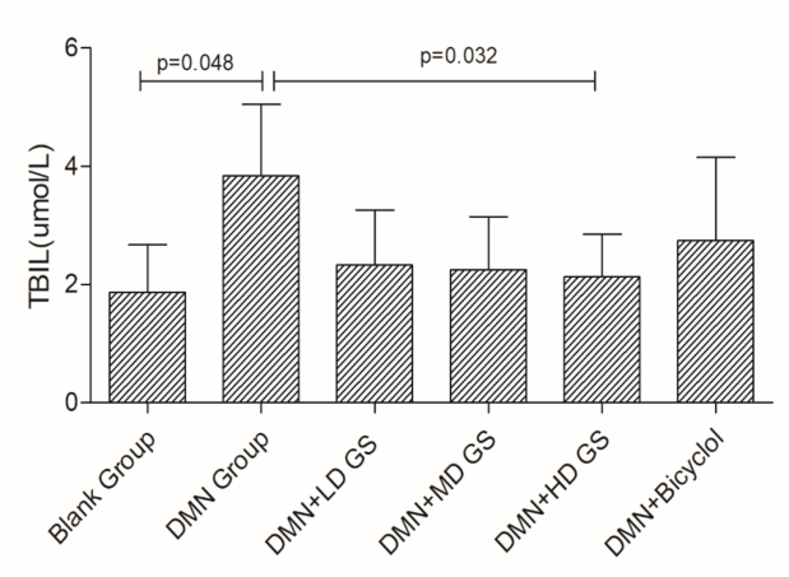

Supplement: S1 File — (ZIP) [file pone.0304185.s001.zip › Single Image-TIF-PACE/Fig1/Fig1C.tif]

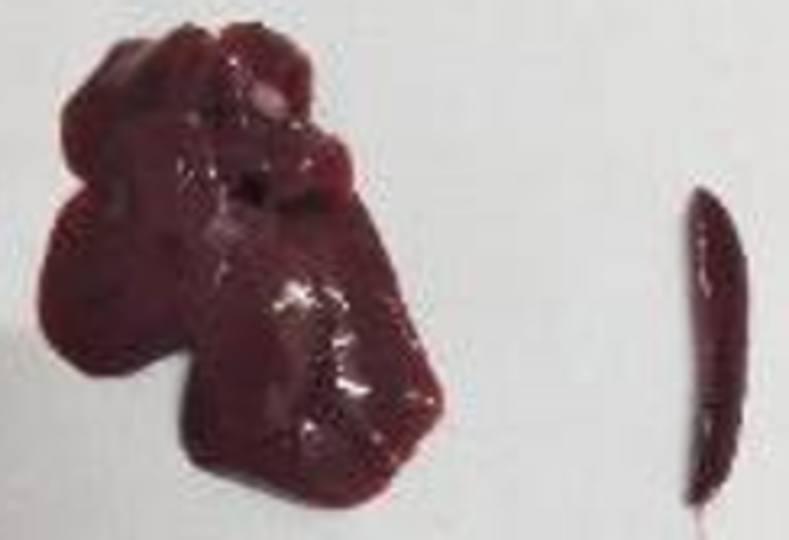

Supplement: S1 File — (ZIP) [file pone.0304185.s001.zip › Single Image-TIF-PACE/Fig2/Fig2A.tif]

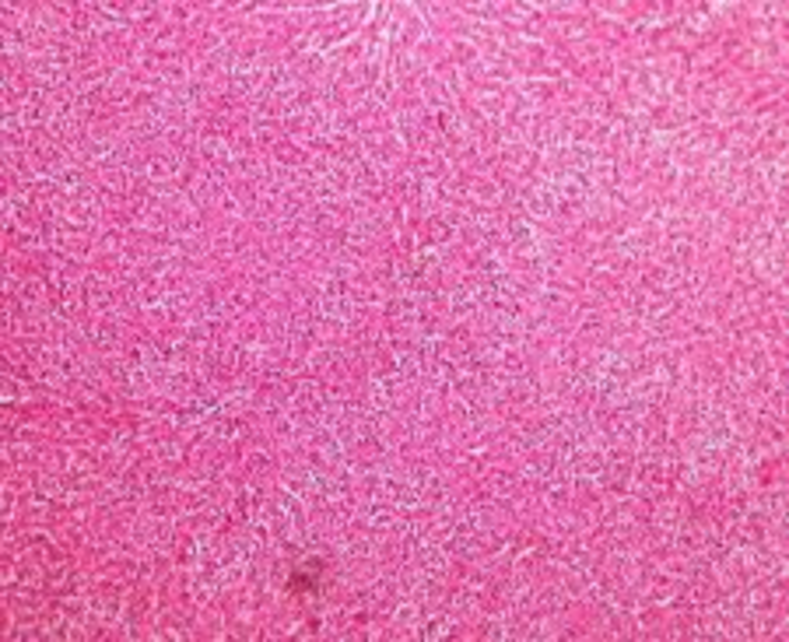

Supplement: S1 File — (ZIP) [file pone.0304185.s001.zip › Single Image-TIF-PACE/Fig2/Fig2A1.tif]

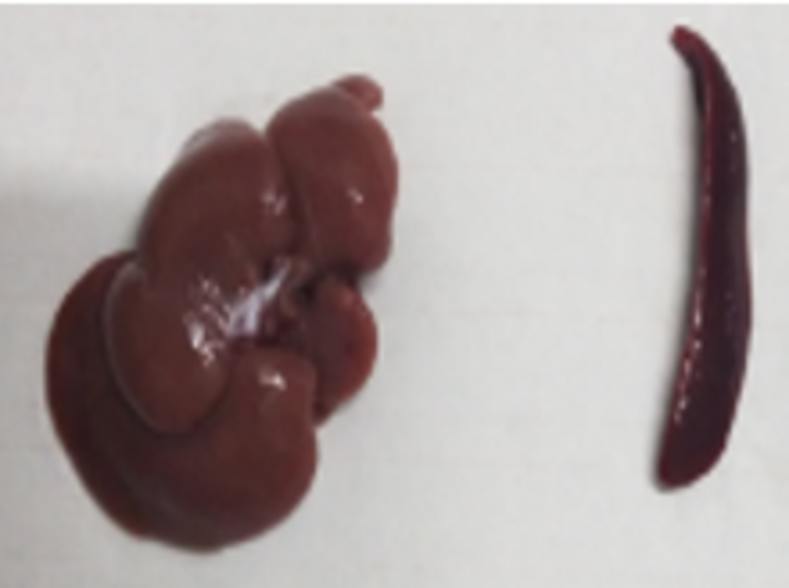

Supplement: S1 File — (ZIP) [file pone.0304185.s001.zip › Single Image-TIF-PACE/Fig2/Fig2B.tif]

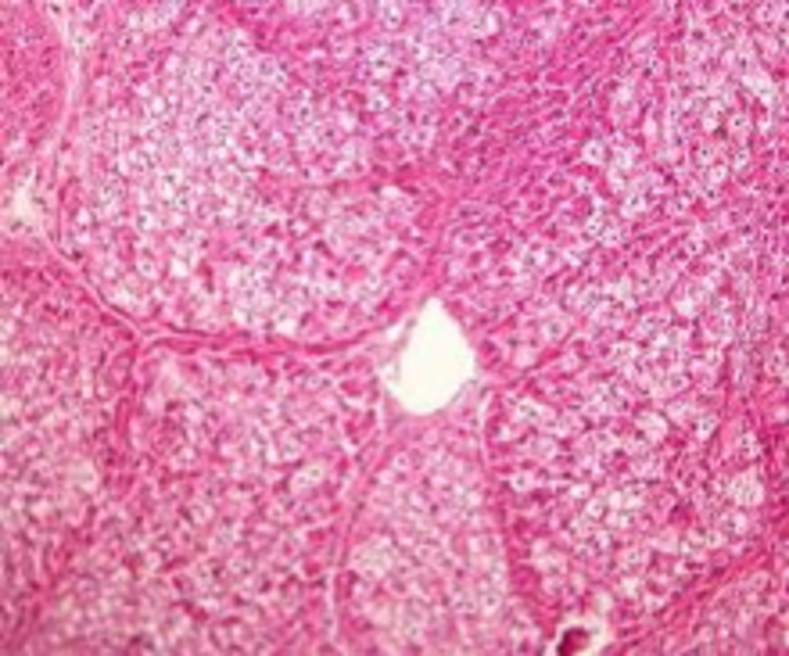

Supplement: S1 File — (ZIP) [file pone.0304185.s001.zip › Single Image-TIF-PACE/Fig2/Fig2B1.tif]

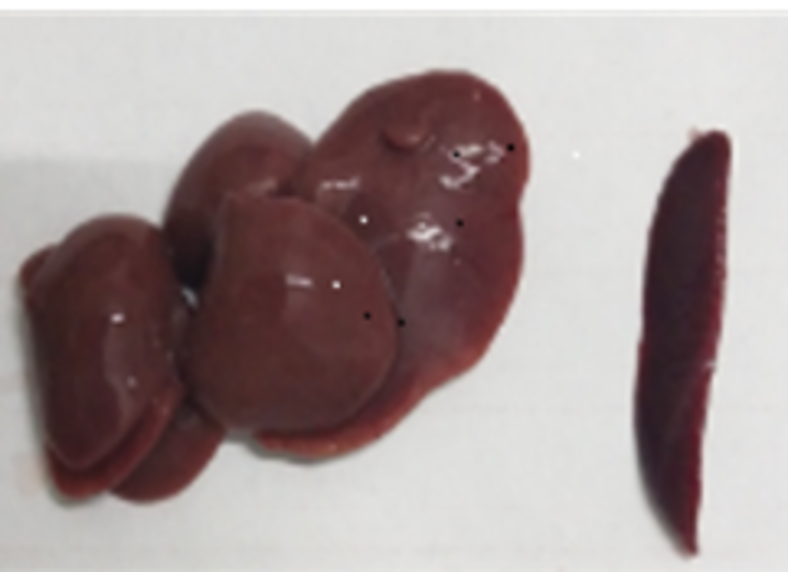

Supplement: S1 File — (ZIP) [file pone.0304185.s001.zip › Single Image-TIF-PACE/Fig2/Fig2C.tif]

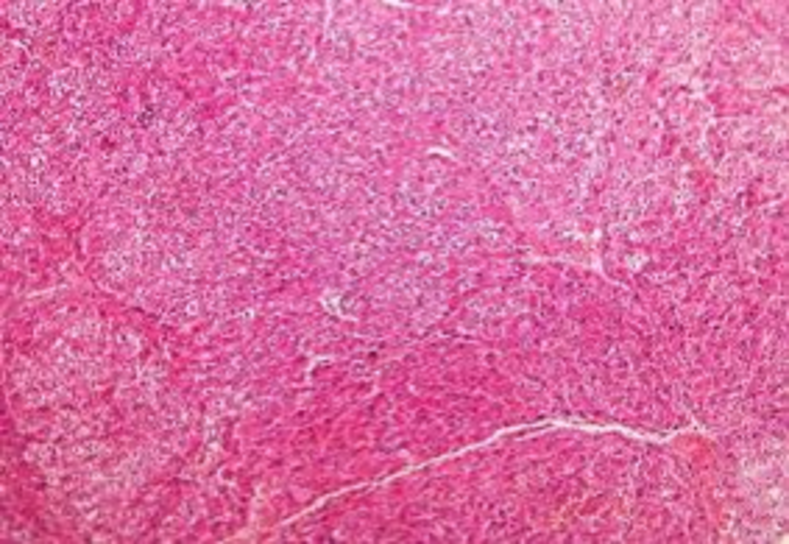

Supplement: S1 File — (ZIP) [file pone.0304185.s001.zip › Single Image-TIF-PACE/Fig2/Fig2C1.tif]

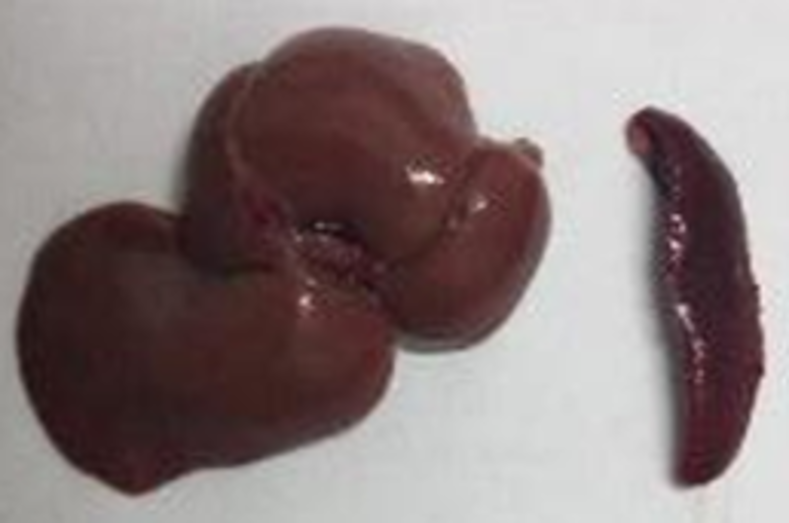

Supplement: S1 File — (ZIP) [file pone.0304185.s001.zip › Single Image-TIF-PACE/Fig2/Fig2D.tif]

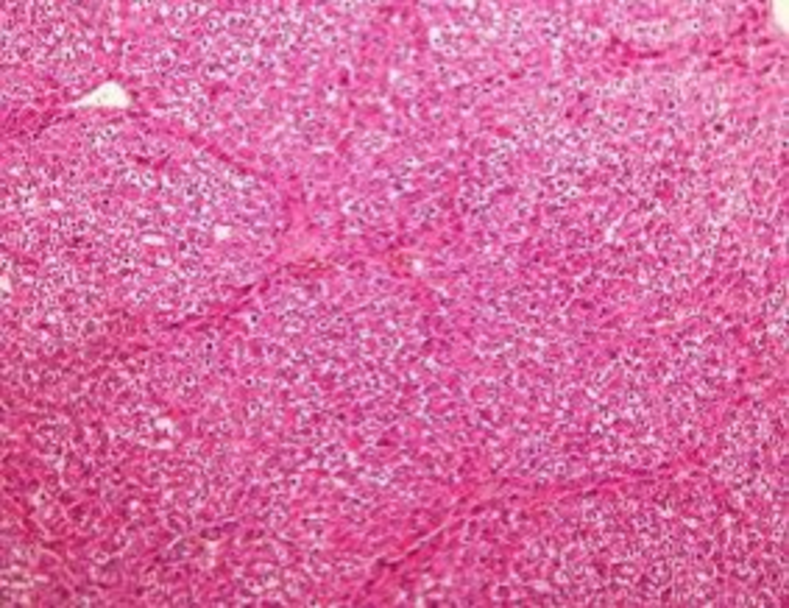

Supplement: S1 File — (ZIP) [file pone.0304185.s001.zip › Single Image-TIF-PACE/Fig2/Fig2D1.tif]

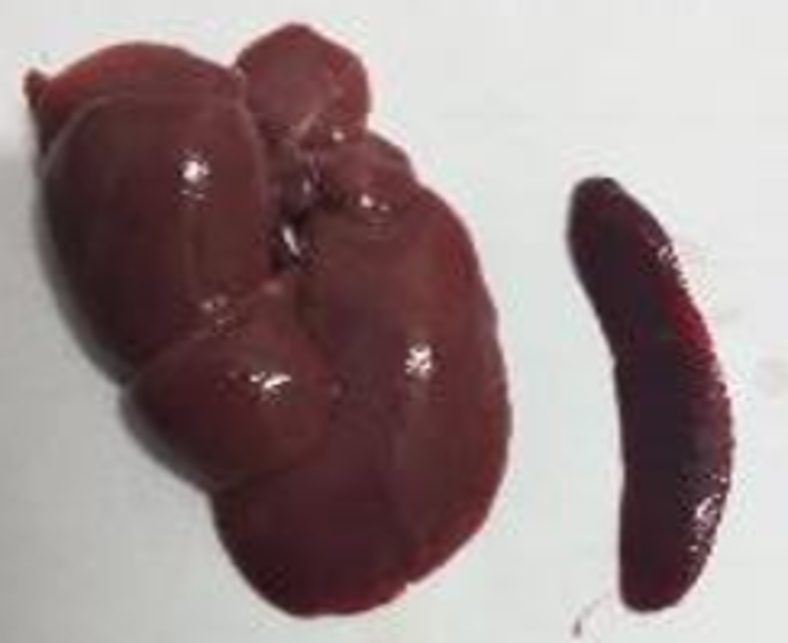

Supplement: S1 File — (ZIP) [file pone.0304185.s001.zip › Single Image-TIF-PACE/Fig2/Fig2E.tif]

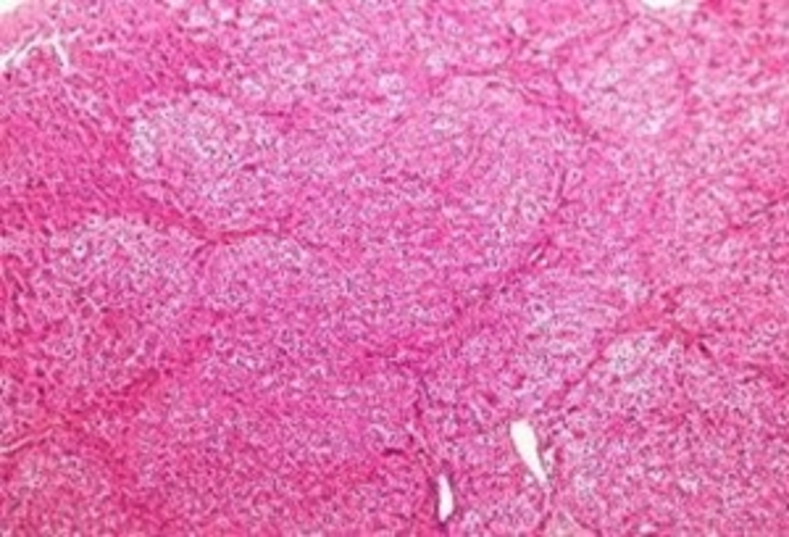

Supplement: S1 File — (ZIP) [file pone.0304185.s001.zip › Single Image-TIF-PACE/Fig2/Fig2E1.tif]

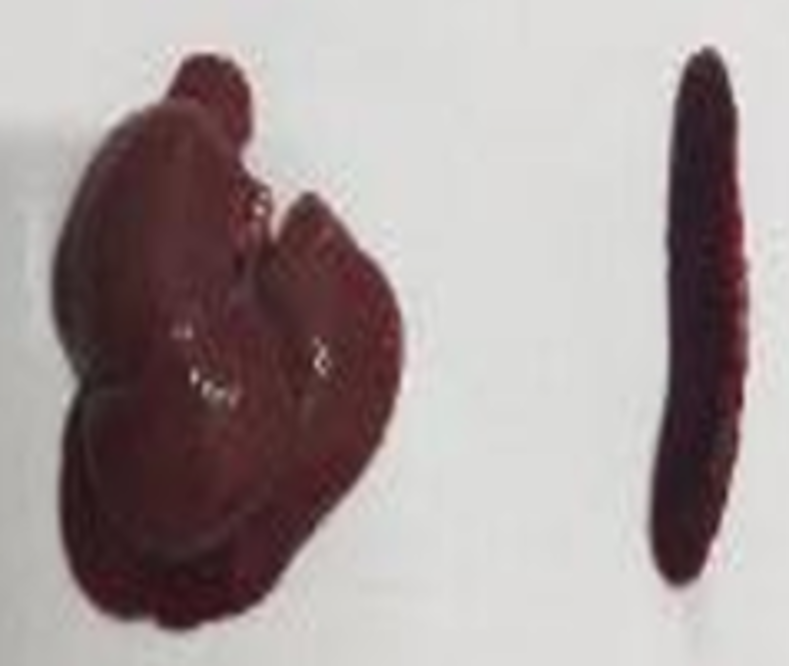

Supplement: S1 File — (ZIP) [file pone.0304185.s001.zip › Single Image-TIF-PACE/Fig2/Fig2F.tif]

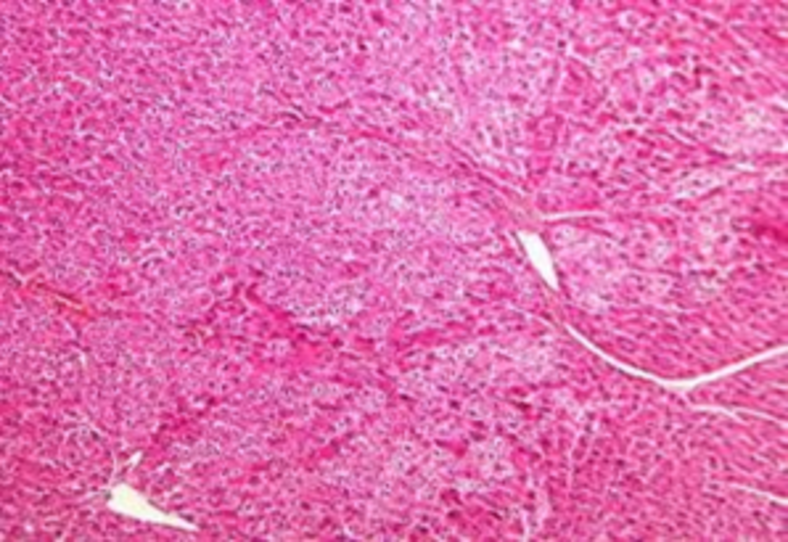

Supplement: S1 File — (ZIP) [file pone.0304185.s001.zip › Single Image-TIF-PACE/Fig2/Fig2F1.tif]

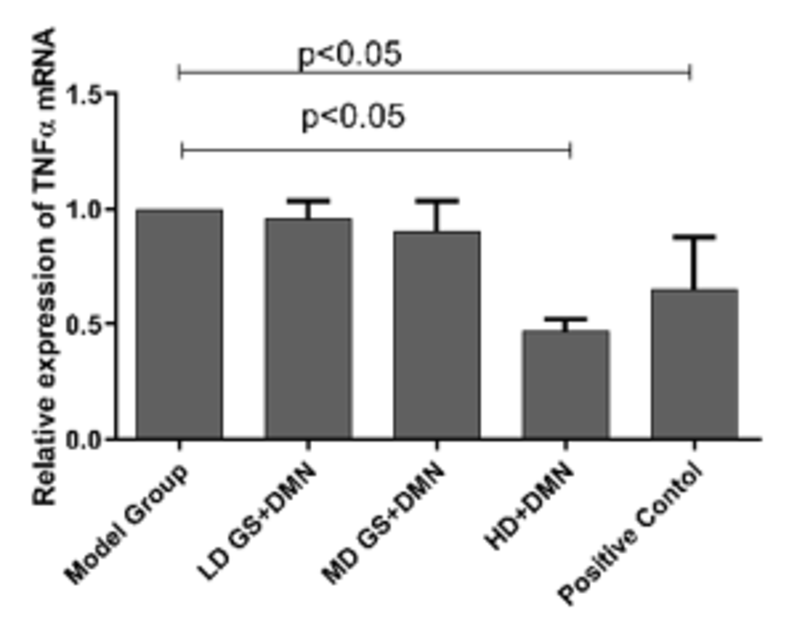

Supplement: S1 File — (ZIP) [file pone.0304185.s001.zip › Single Image-TIF-PACE/Fig3/Fig3A.tif]

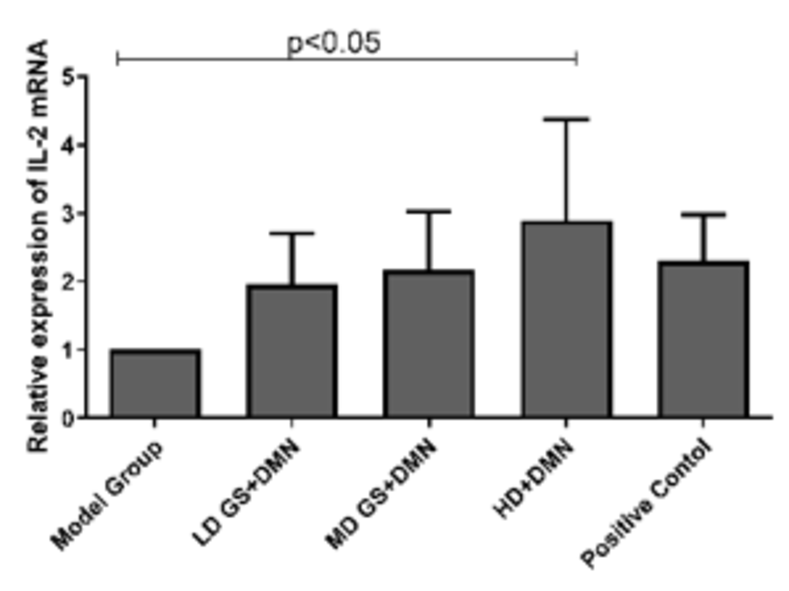

Supplement: S1 File — (ZIP) [file pone.0304185.s001.zip › Single Image-TIF-PACE/Fig3/Fig3B.tif]

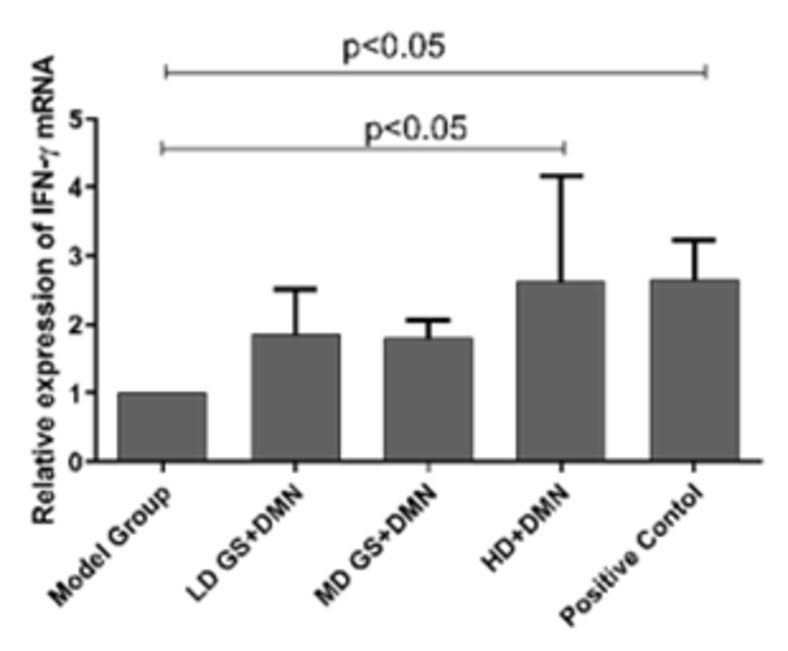

Supplement: S1 File — (ZIP) [file pone.0304185.s001.zip › Single Image-TIF-PACE/Fig3/Fig3C.tif]

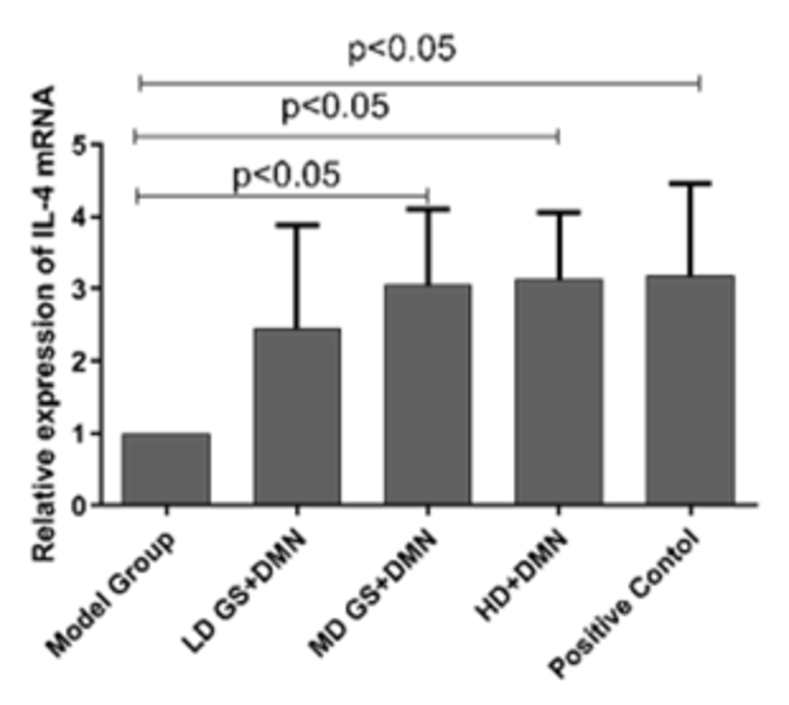

Supplement: S1 File — (ZIP) [file pone.0304185.s001.zip › Single Image-TIF-PACE/Fig3/Fig3D.tif]

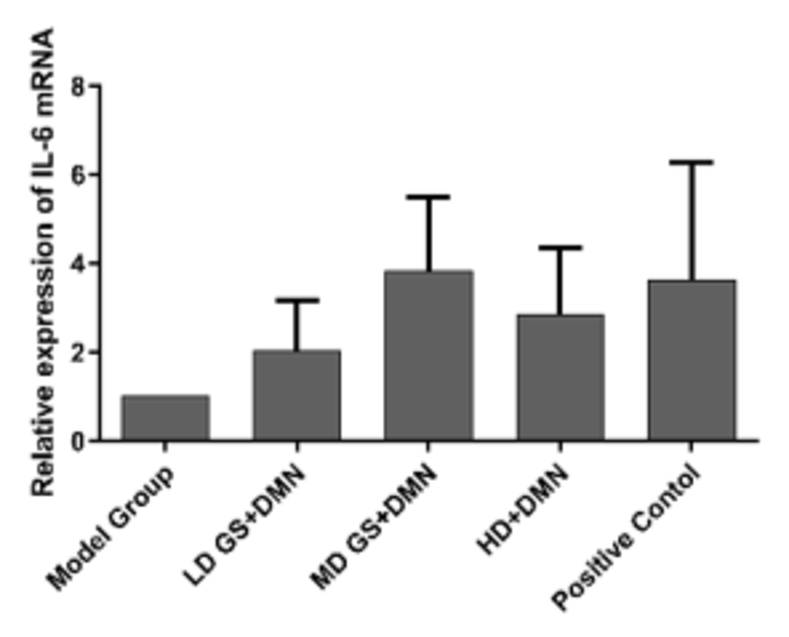

Supplement: S1 File — (ZIP) [file pone.0304185.s001.zip › Single Image-TIF-PACE/Fig3/Fig3E.tif]

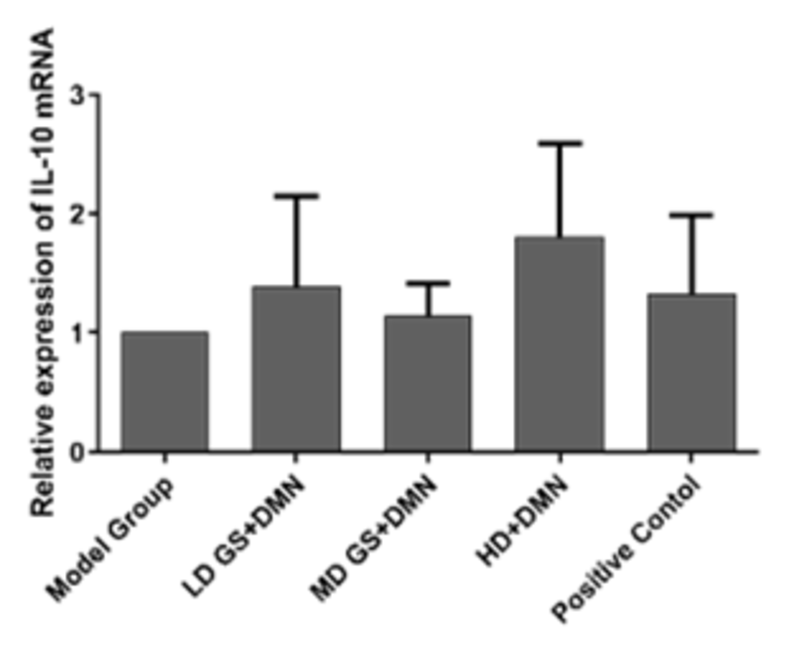

Supplement: S1 File — (ZIP) [file pone.0304185.s001.zip › Single Image-TIF-PACE/Fig3/Fig3F.tif]

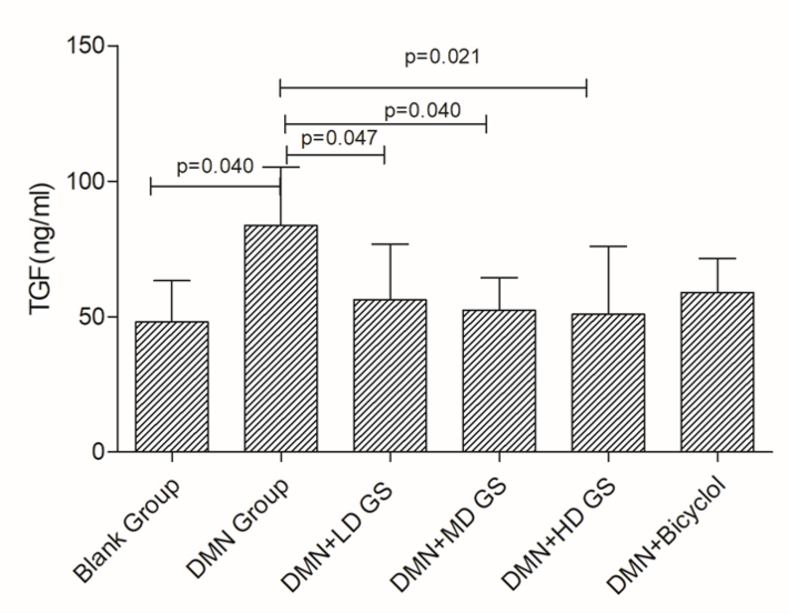

Supplement: S1 File — (ZIP) [file pone.0304185.s001.zip › Single Image-TIF-PACE/Fig4/Fig4A.tif]

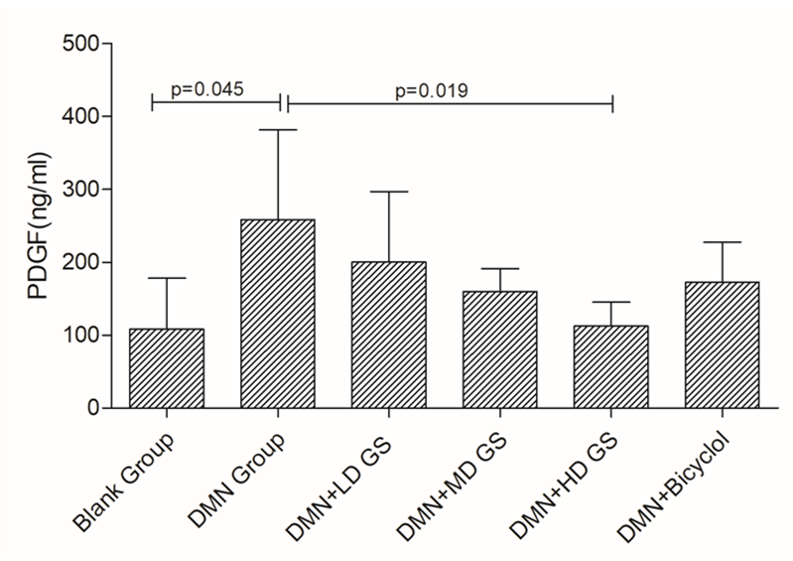

Supplement: S1 File — (ZIP) [file pone.0304185.s001.zip › Single Image-TIF-PACE/Fig4/Fig4B.tif]

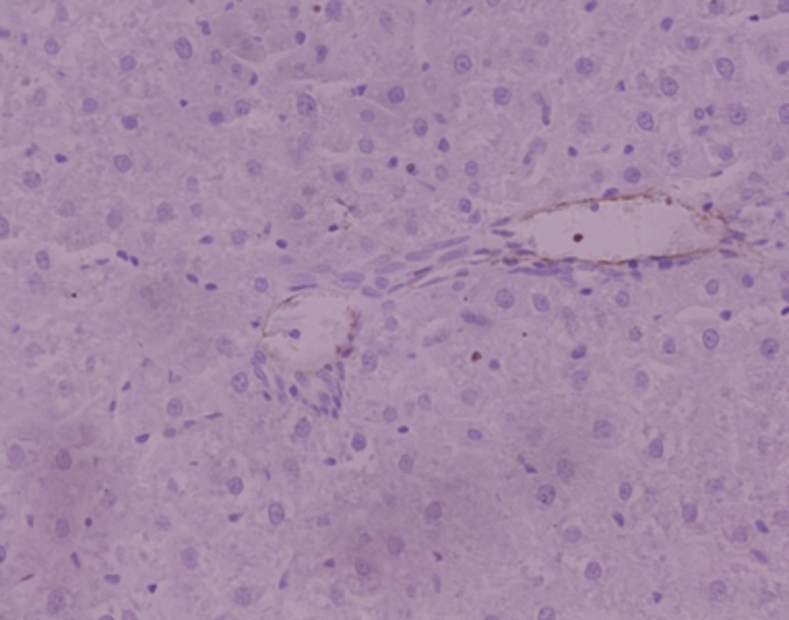

Supplement: S1 File — (ZIP) [file pone.0304185.s001.zip › Single Image-TIF-PACE/Fig4/Fig4C.tif]

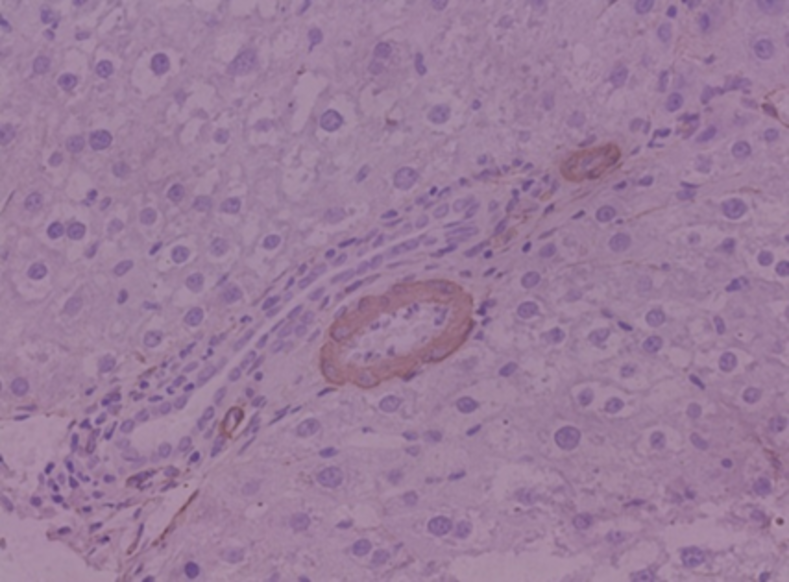

Supplement: S1 File — (ZIP) [file pone.0304185.s001.zip › Single Image-TIF-PACE/Fig4/Fig4D.tif]

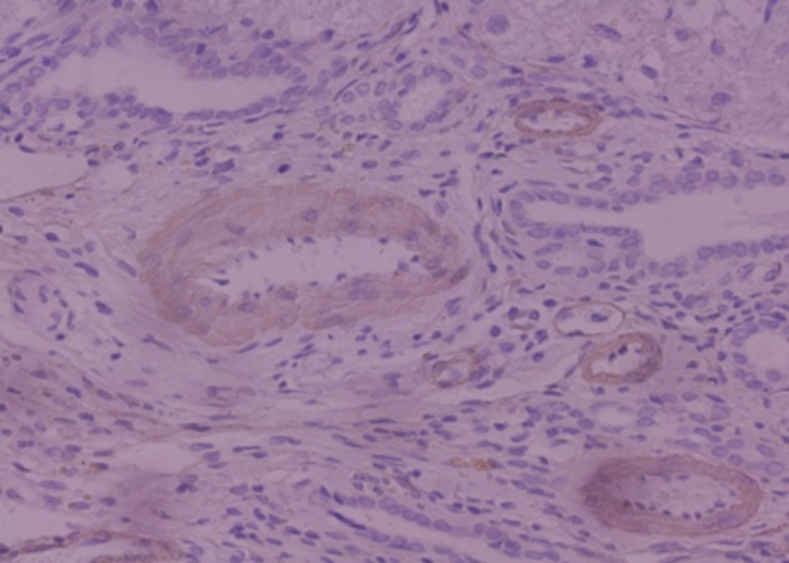

Supplement: S1 File — (ZIP) [file pone.0304185.s001.zip › Single Image-TIF-PACE/Fig4/Fig4E.tif]

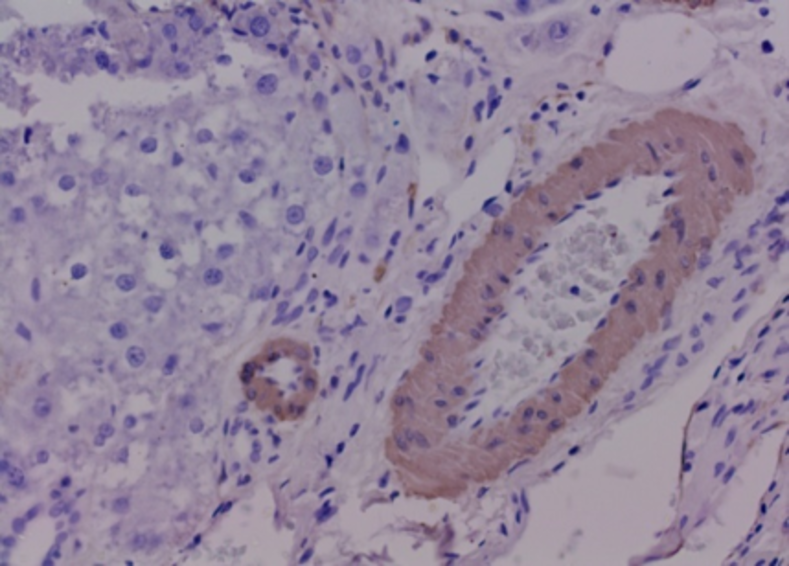

Supplement: S1 File — (ZIP) [file pone.0304185.s001.zip › Single Image-TIF-PACE/Fig4/Fig4F.tif]

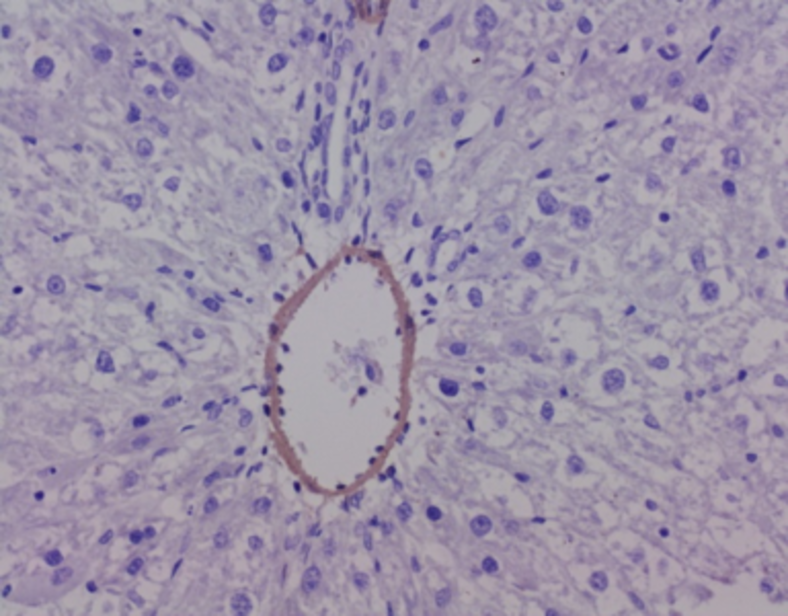

Supplement: S1 File — (ZIP) [file pone.0304185.s001.zip › Single Image-TIF-PACE/Fig4/Fig4G.tif]

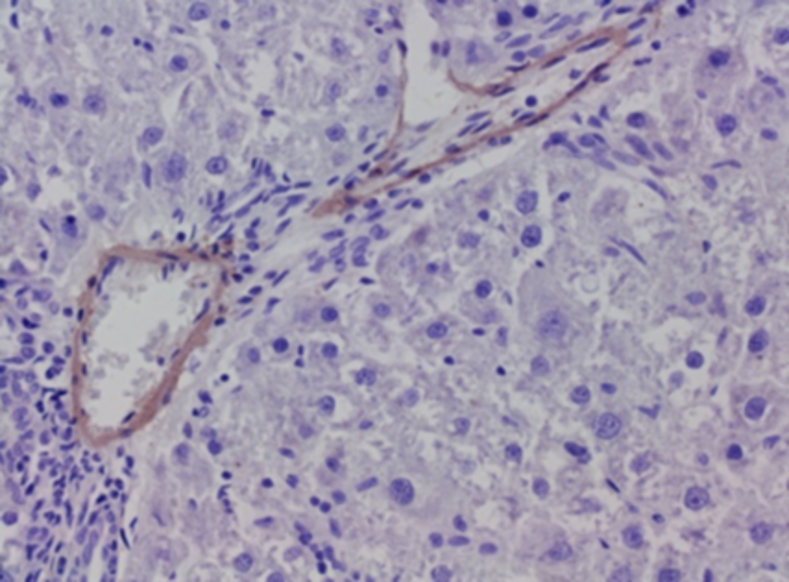

Supplement: S1 File — (ZIP) [file pone.0304185.s001.zip › Single Image-TIF-PACE/Fig4/Fig4H.tif]

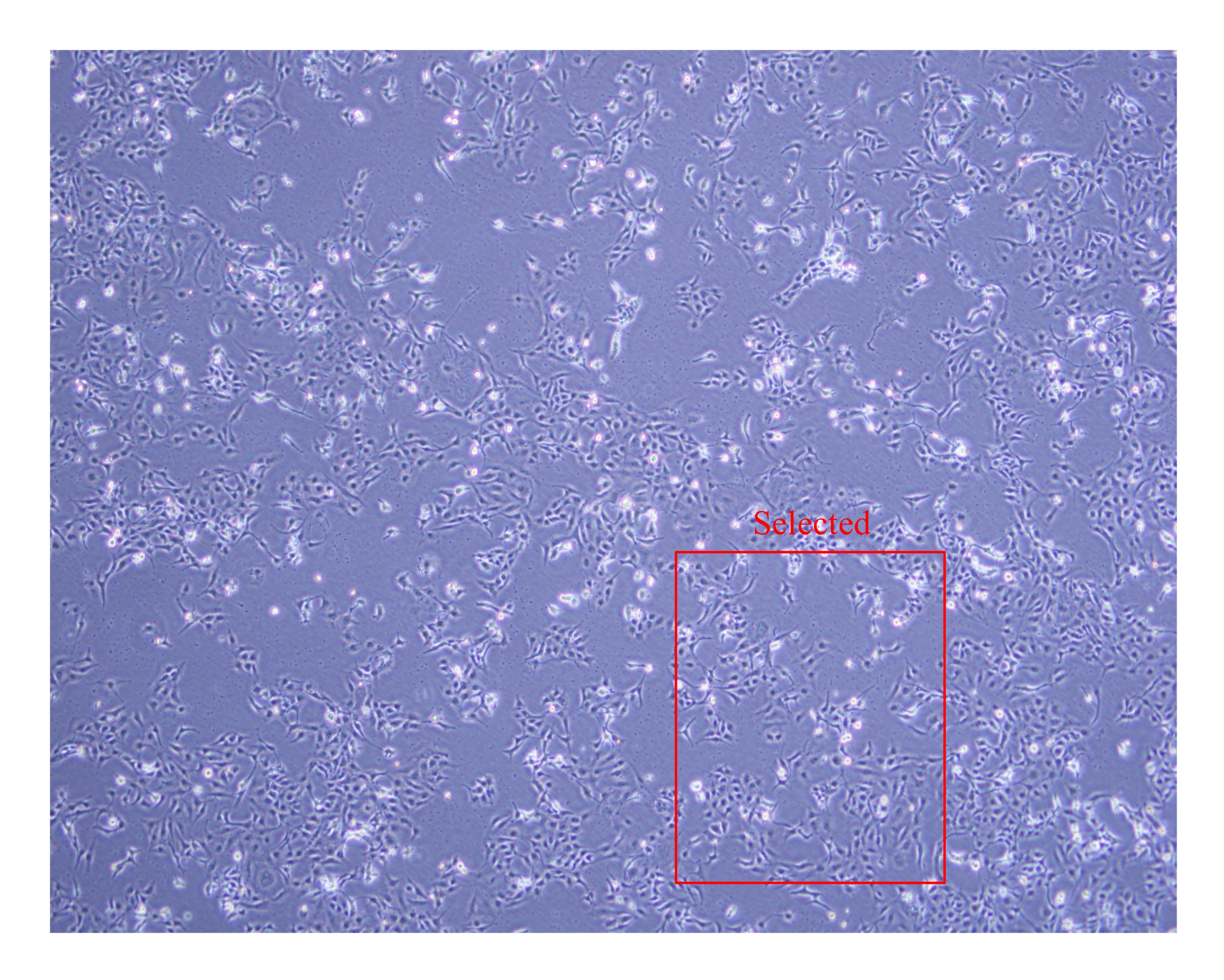

Supplement: S1 File — (ZIP) [file pone.0304185.s001.zip › Single Image-TIF-PACE/Fig5/Fig5A-Large image-HSCs Normal control-4x.tif]

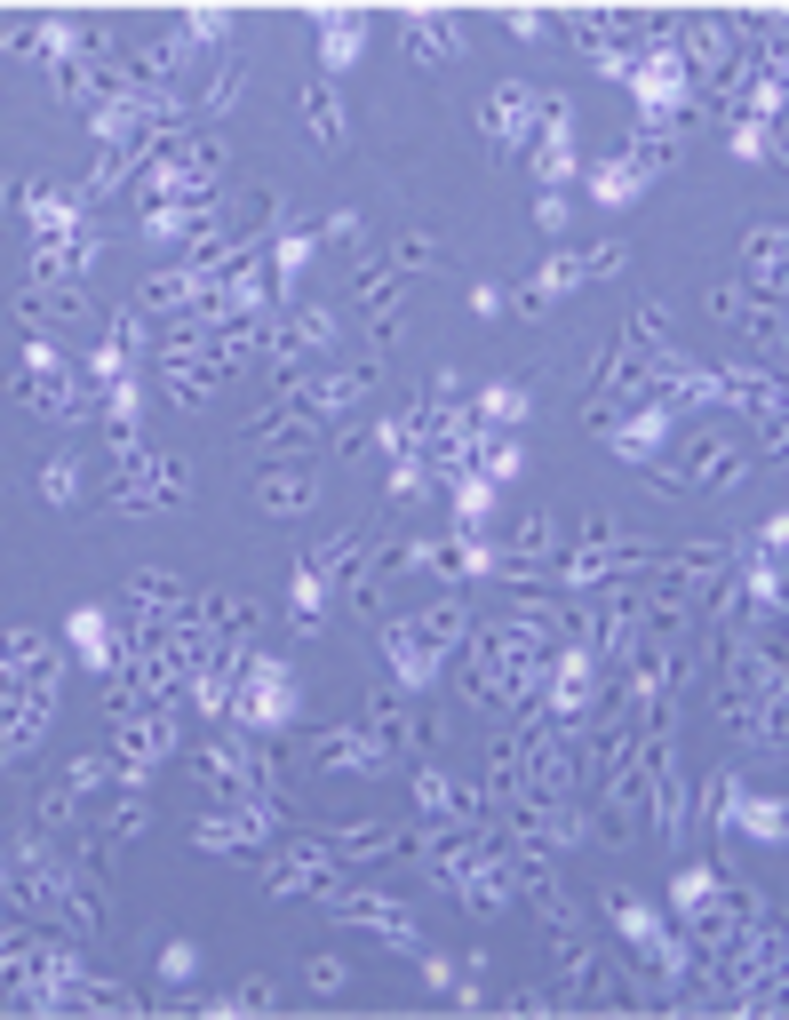

Supplement: S1 File — (ZIP) [file pone.0304185.s001.zip › Single Image-TIF-PACE/Fig5/Fig5A.tif]

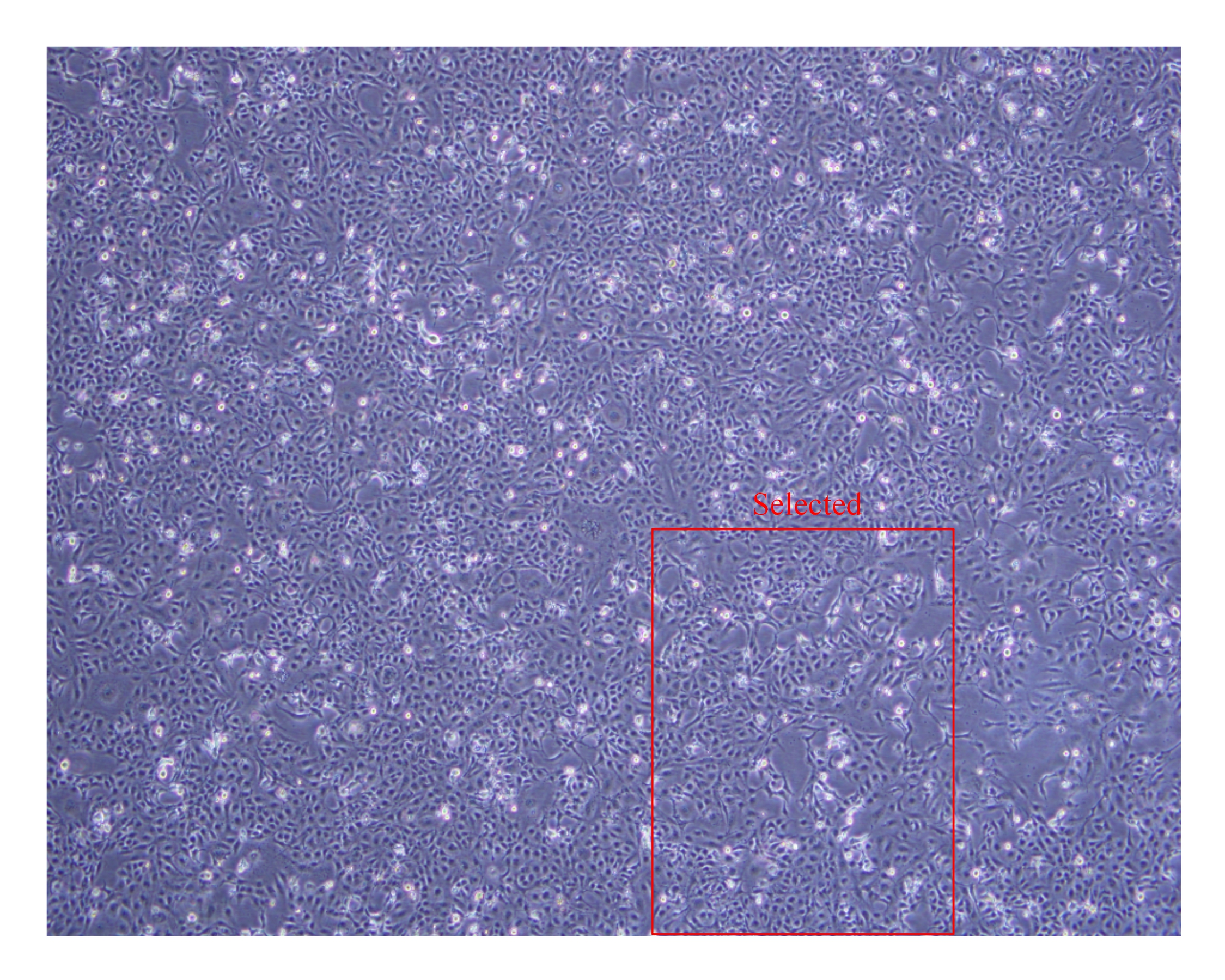

Supplement: S1 File — (ZIP) [file pone.0304185.s001.zip › Single Image-TIF-PACE/Fig5/Fig5B-Large image-HSCs+TNF-4x.tif]

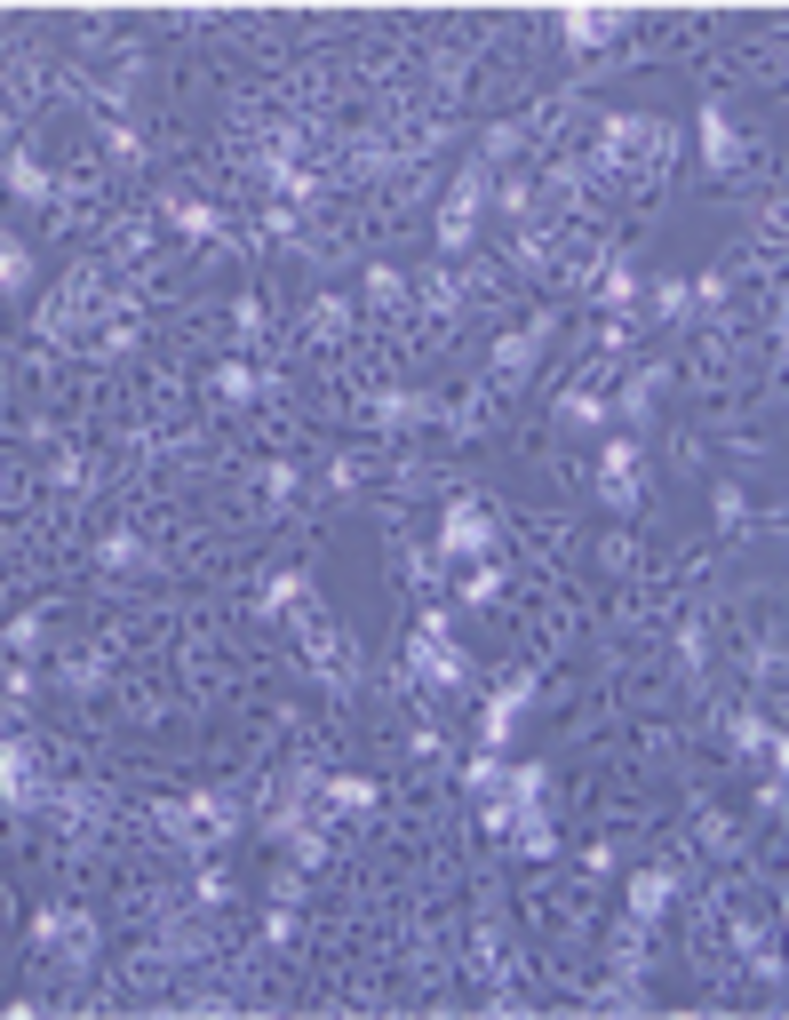

Supplement: S1 File — (ZIP) [file pone.0304185.s001.zip › Single Image-TIF-PACE/Fig5/Fig5B.tif]

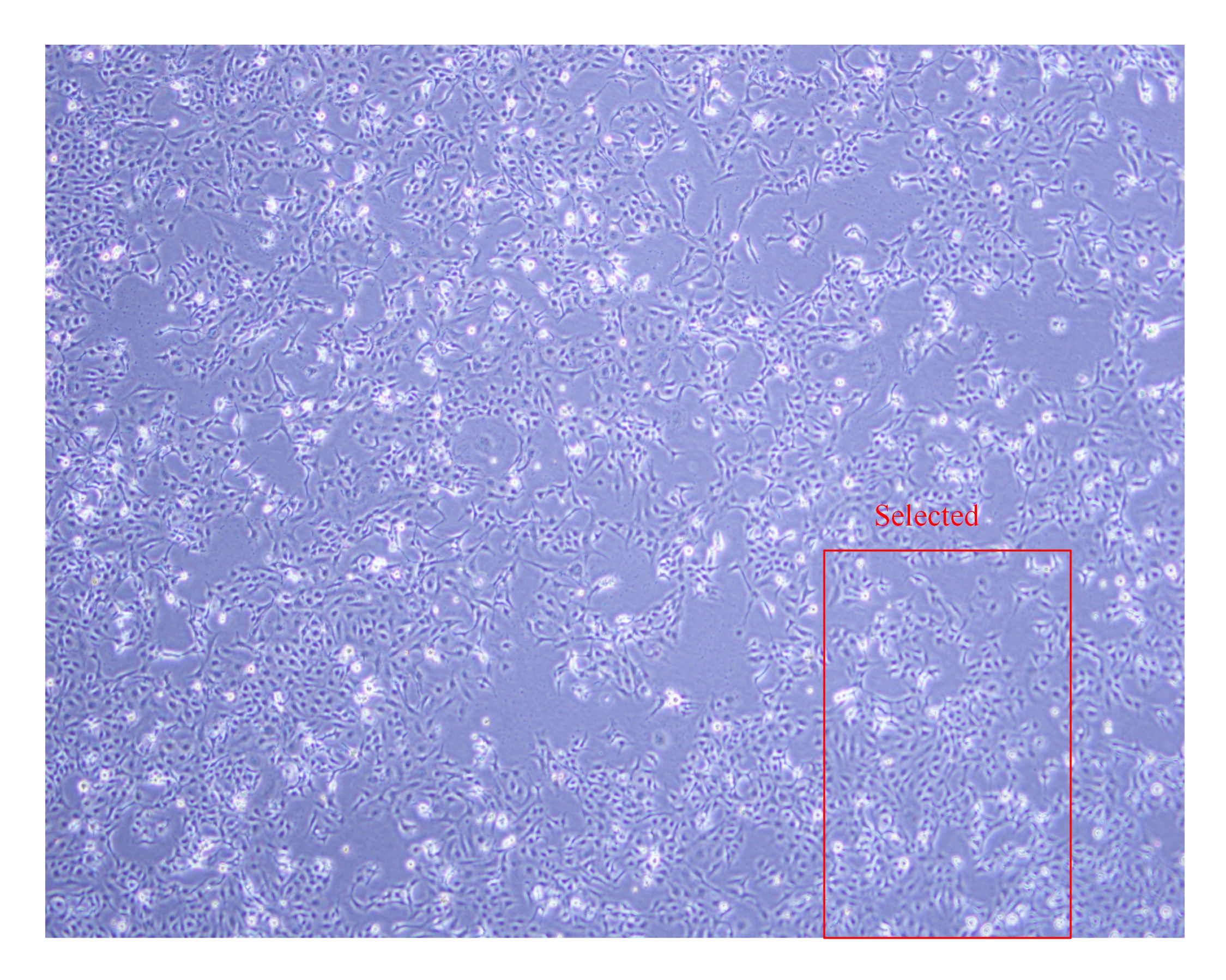

Supplement: S1 File — (ZIP) [file pone.0304185.s001.zip › Single Image-TIF-PACE/Fig5/Fig5C-Large image-HSCs+TNF+naringin-4x.tif]

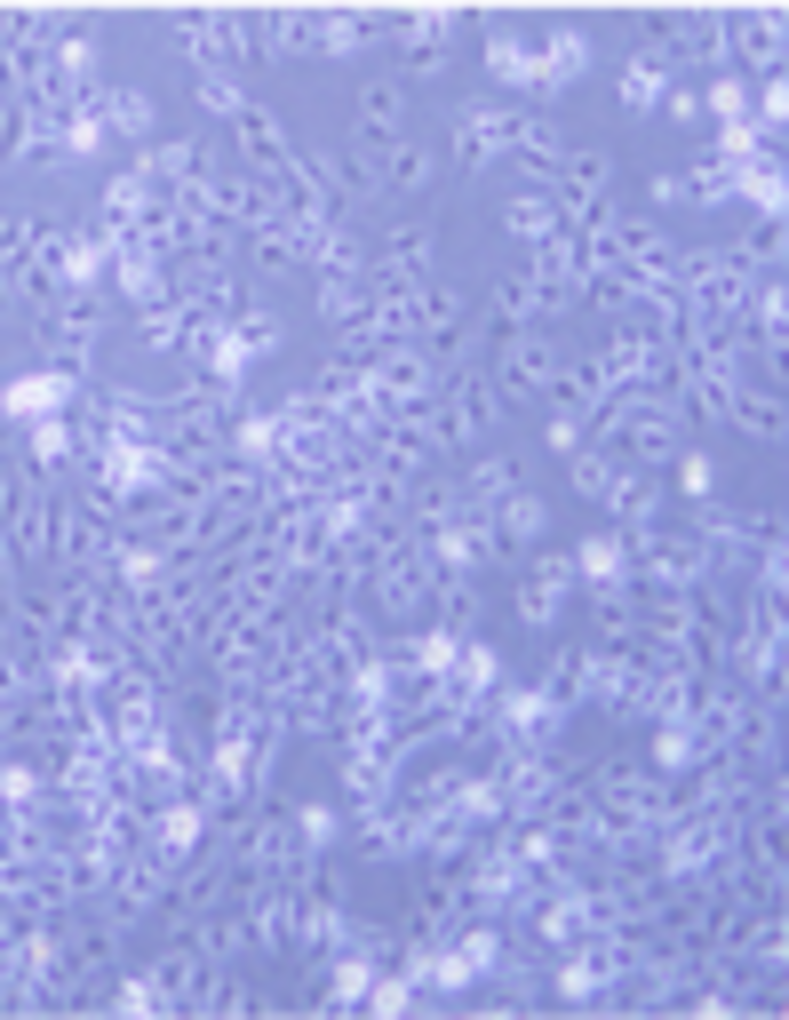

Supplement: S1 File — (ZIP) [file pone.0304185.s001.zip › Single Image-TIF-PACE/Fig5/Fig5C.tif]

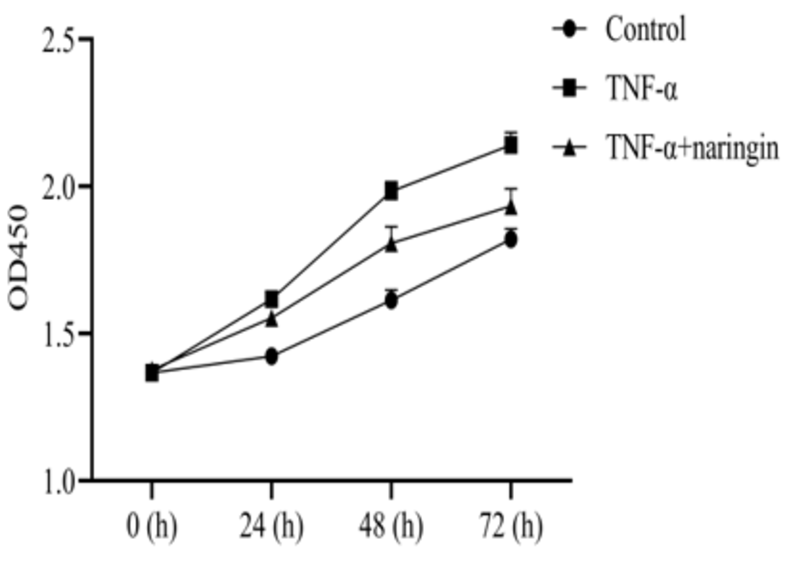

Supplement: S1 File — (ZIP) [file pone.0304185.s001.zip › Single Image-TIF-PACE/Fig5/Fig5D.tif]

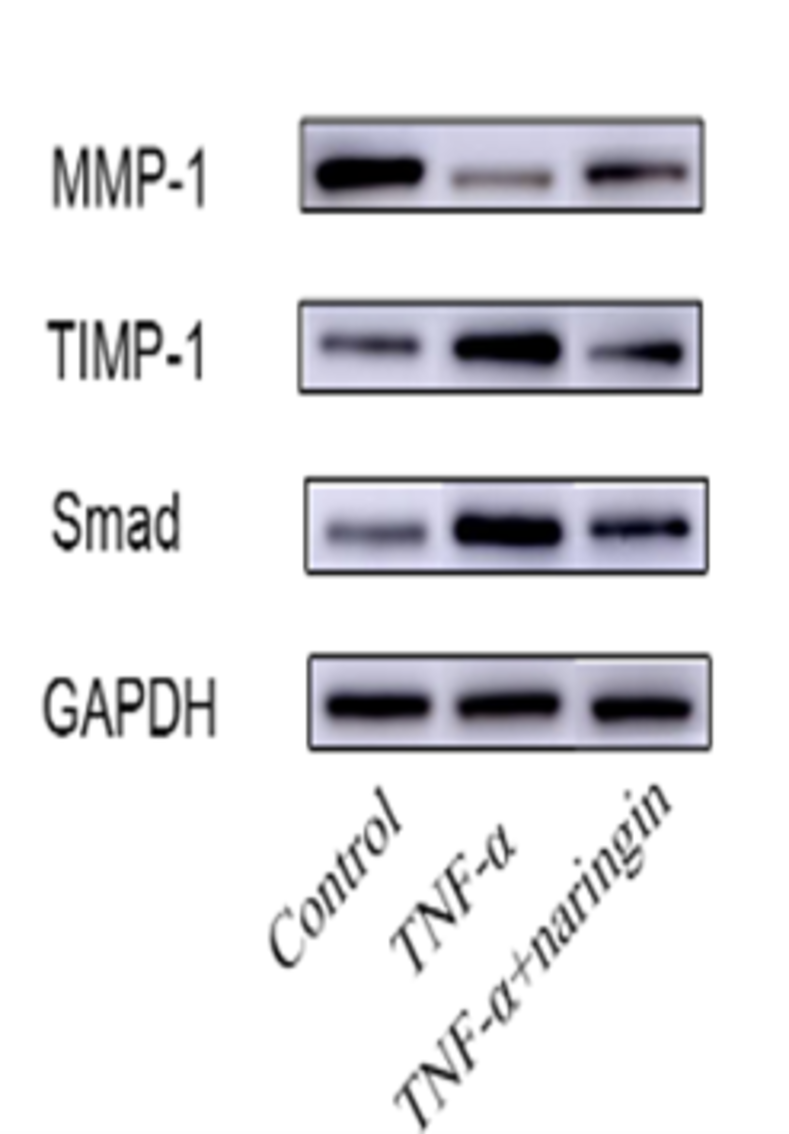

Supplement: S1 File — (ZIP) [file pone.0304185.s001.zip › Single Image-TIF-PACE/Fig5/Fig5E.tif]

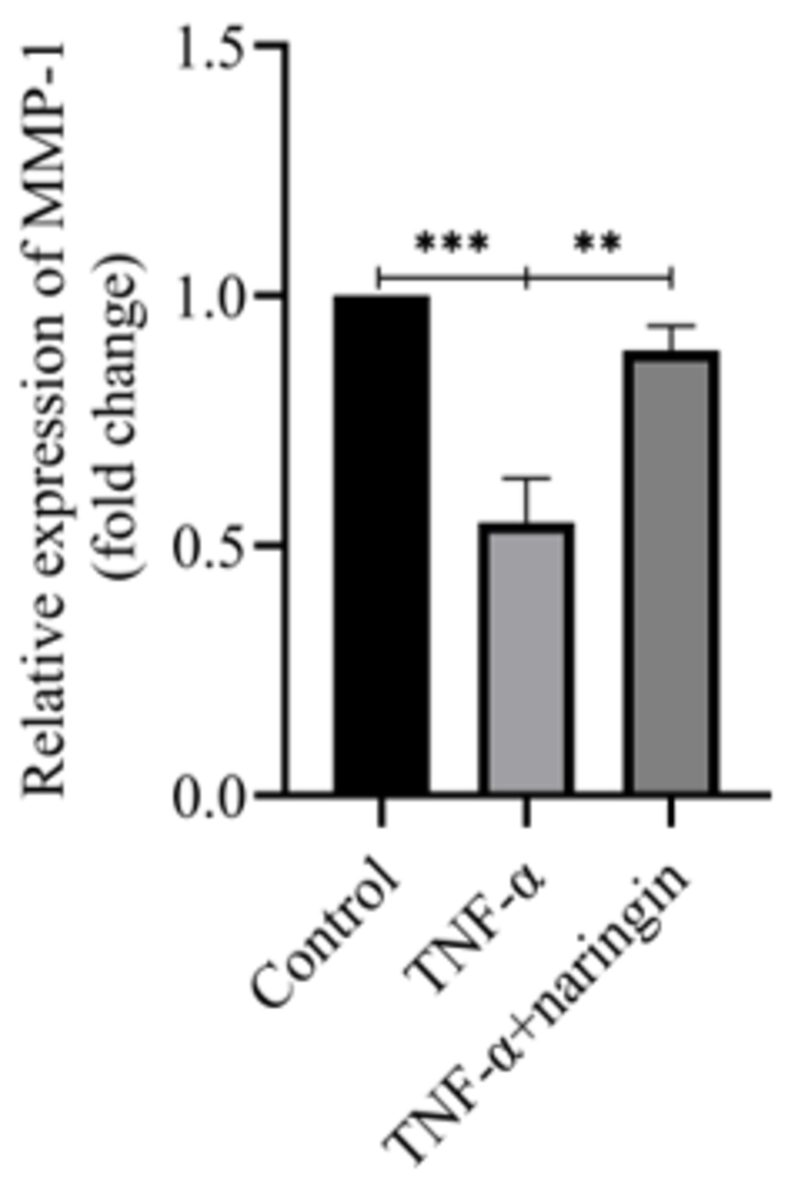

Supplement: S1 File — (ZIP) [file pone.0304185.s001.zip › Single Image-TIF-PACE/Fig5/Fig5F.tif]

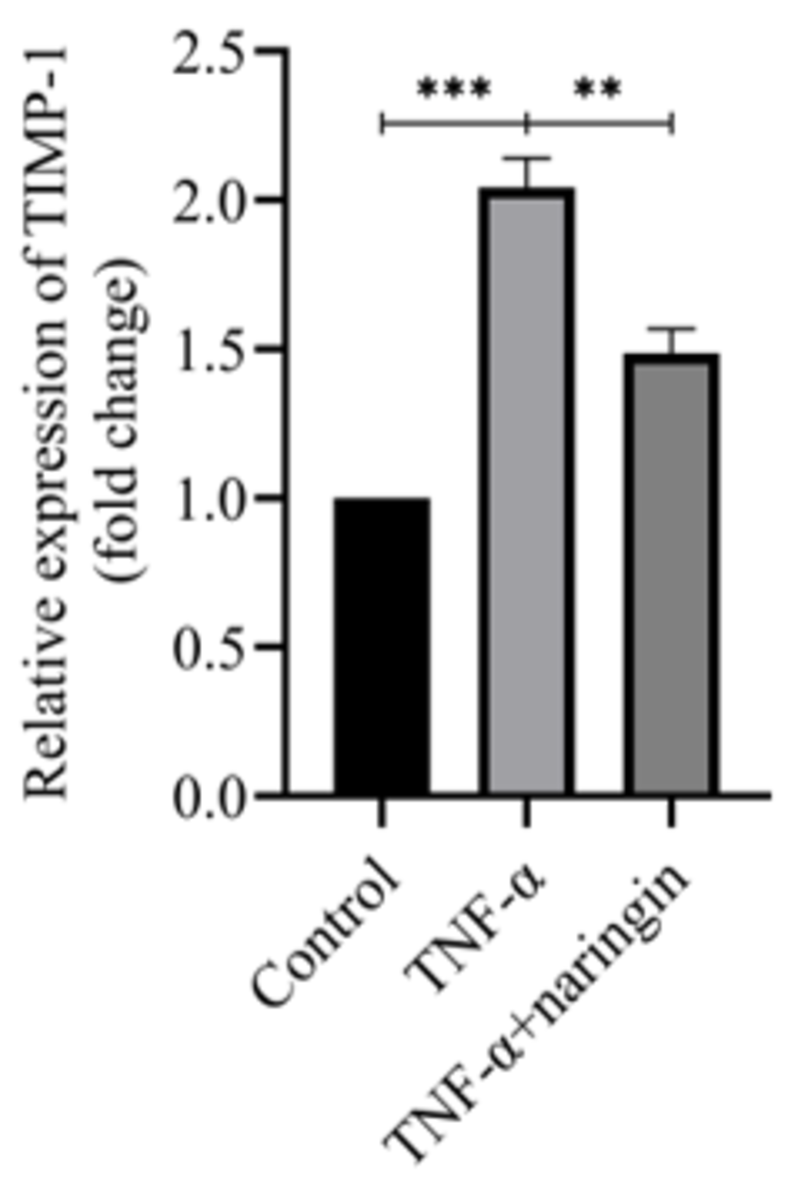

Supplement: S1 File — (ZIP) [file pone.0304185.s001.zip › Single Image-TIF-PACE/Fig5/Fig5G.tif]

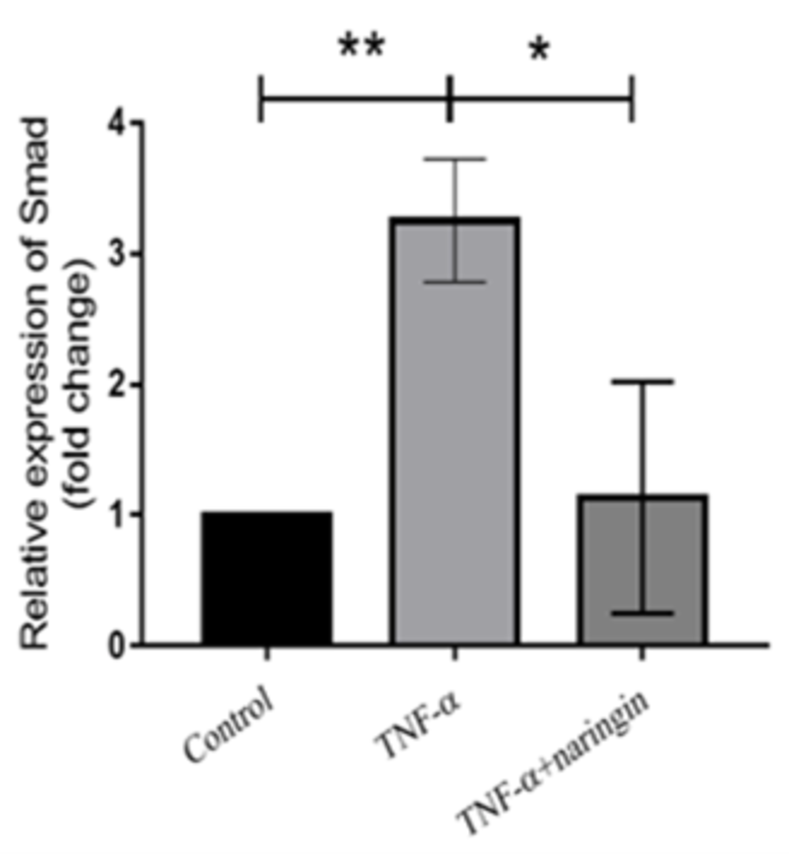

Supplement: S1 File — (ZIP) [file pone.0304185.s001.zip › Single Image-TIF-PACE/Fig5/Fig5H.tif]

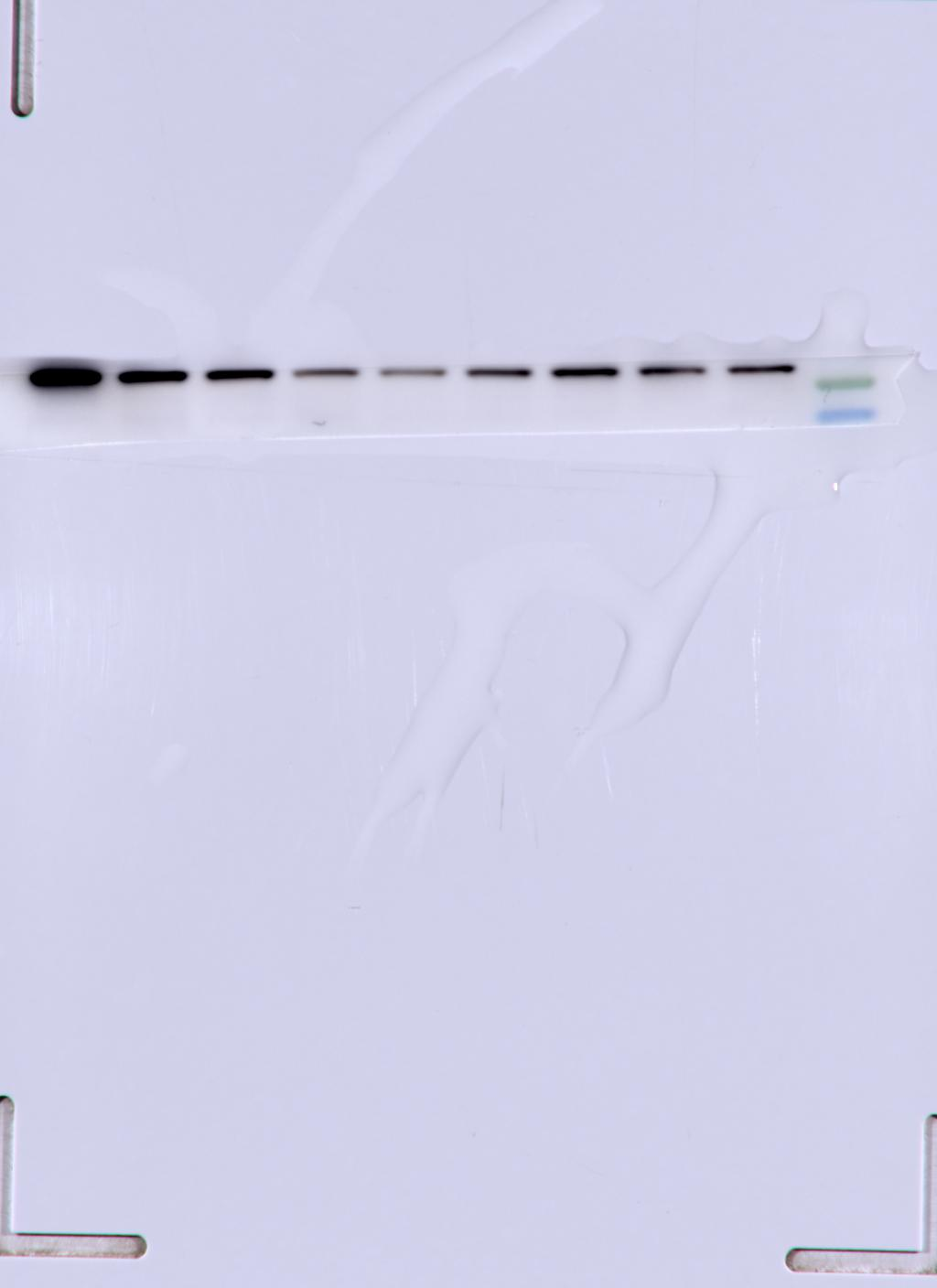

Supplement: S1 File — (ZIP) [file pone.0304185.s001.zip › Single Image-TIF-PACE/Fig5/MMP-1.tif]

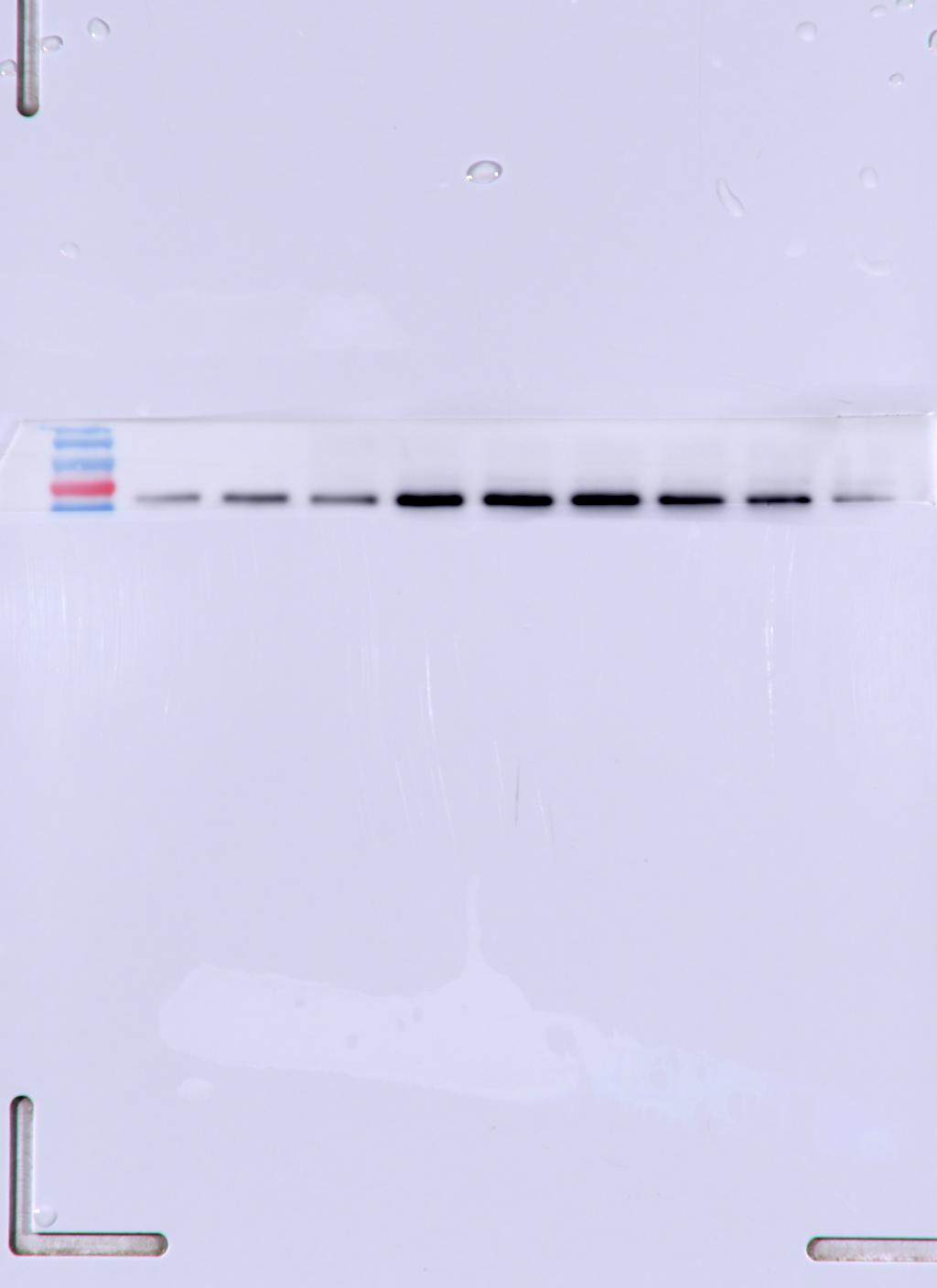

Supplement: S1 File — (ZIP) [file pone.0304185.s001.zip › Single Image-TIF-PACE/Fig5/Smad2.tif]

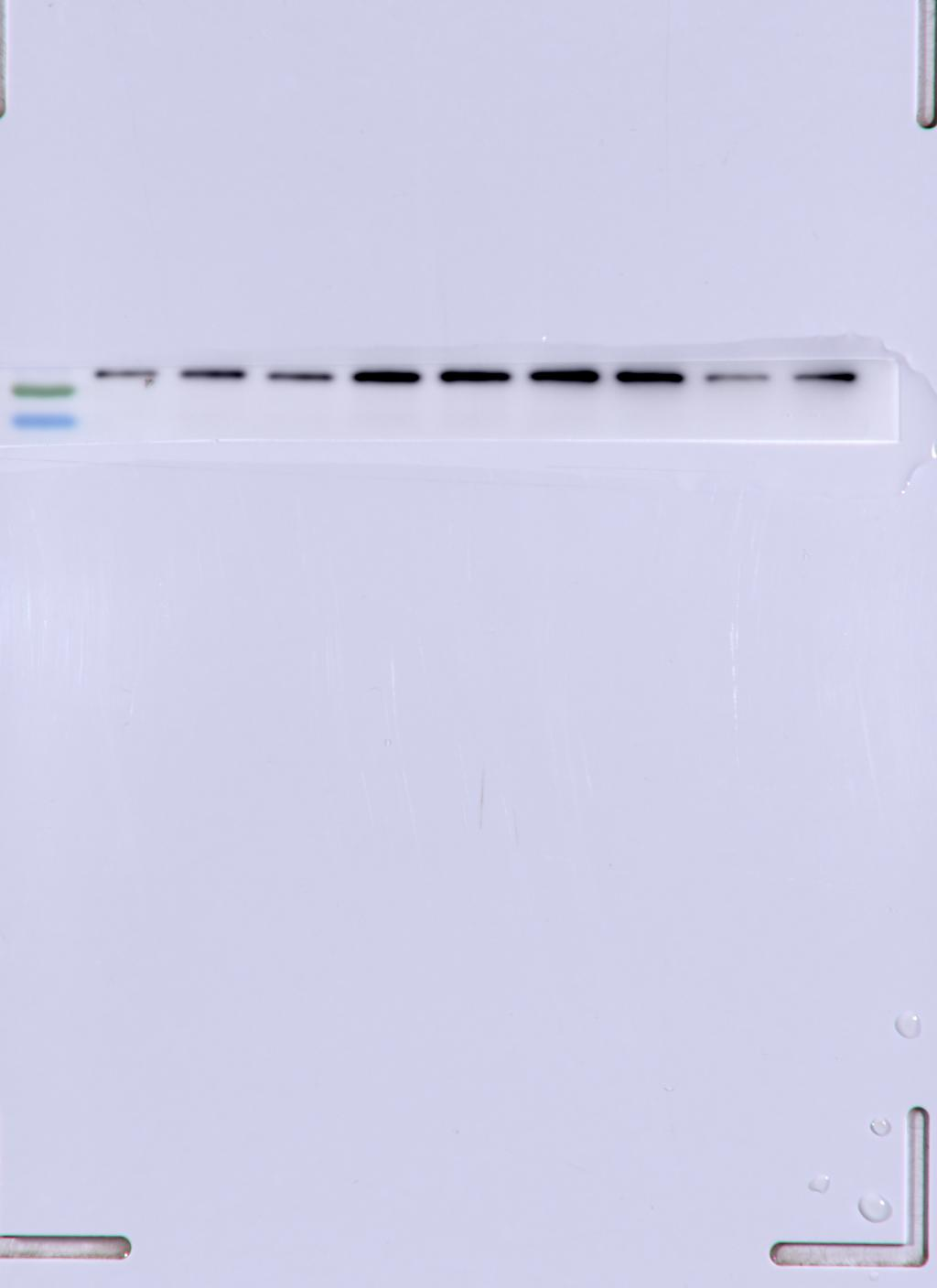

Supplement: S1 File — (ZIP) [file pone.0304185.s001.zip › Single Image-TIF-PACE/Fig5/TIMP-1.tif]

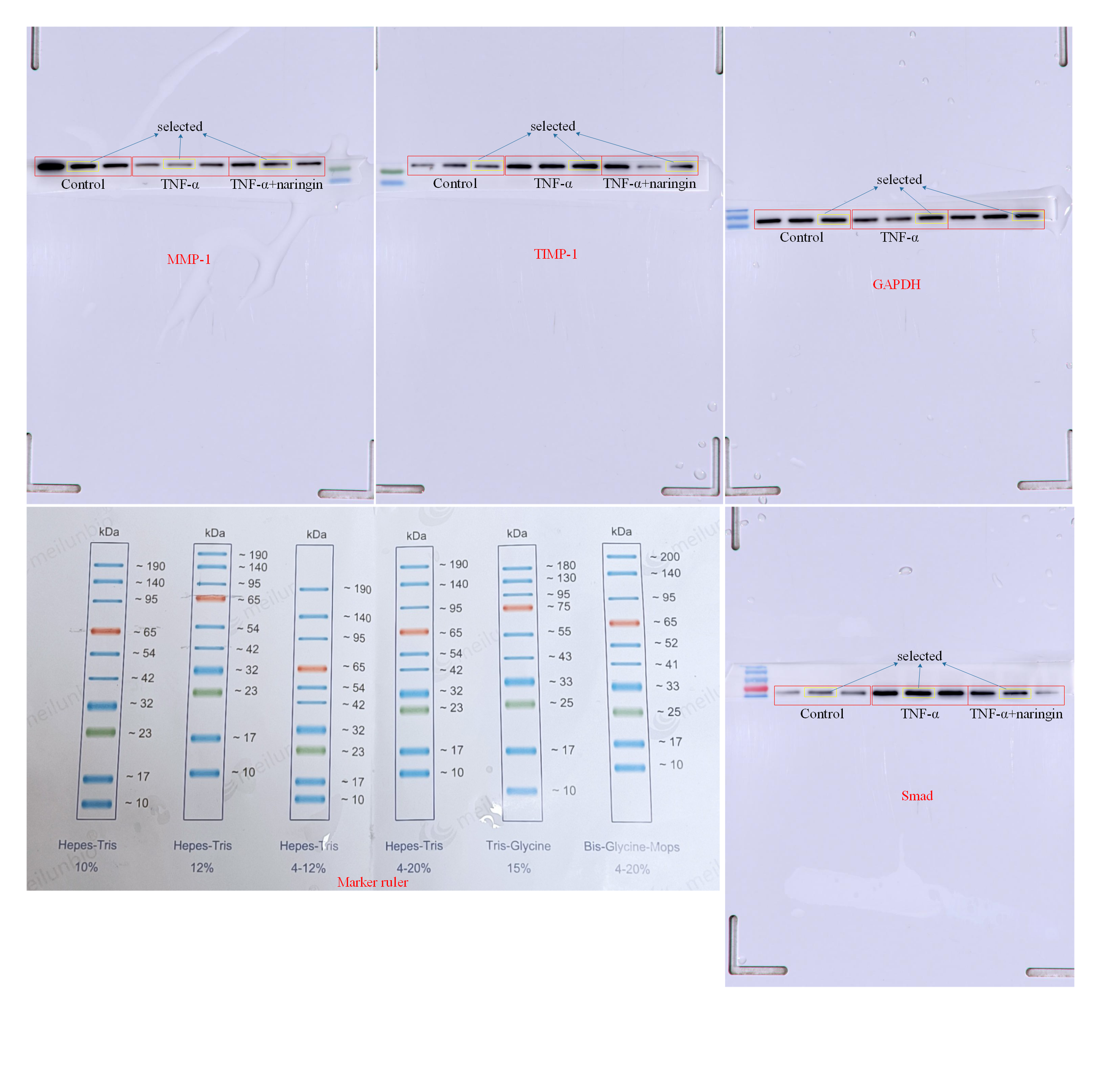

Supplement: S1 File — (ZIP) [file pone.0304185.s001.zip › Single Image-TIF-PACE/Fig5/The original image.tif]

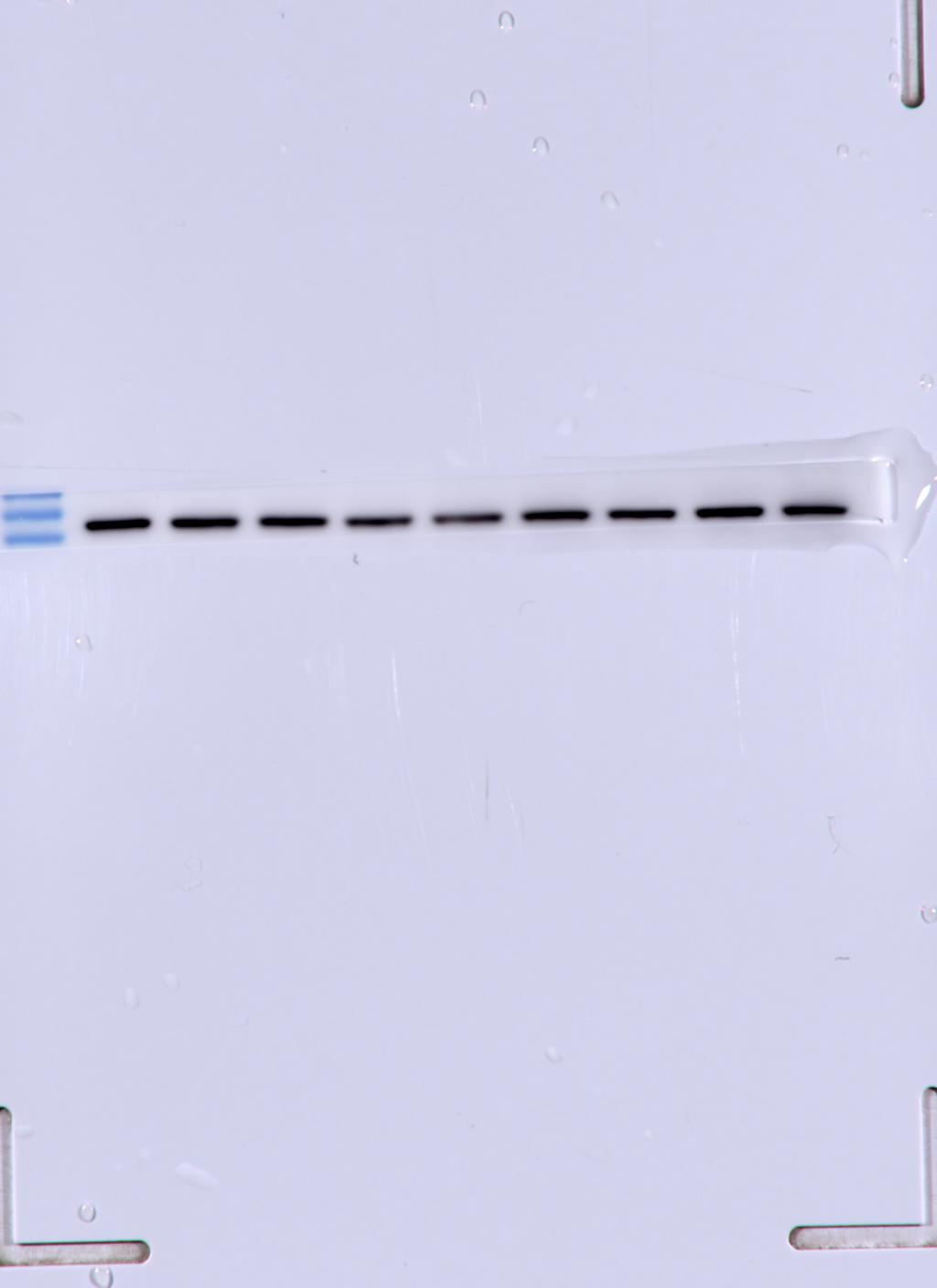

Supplement: S1 File — (ZIP) [file pone.0304185.s001.zip › Single Image-TIF-PACE/Fig5/internal reference-GAPDH.tif]

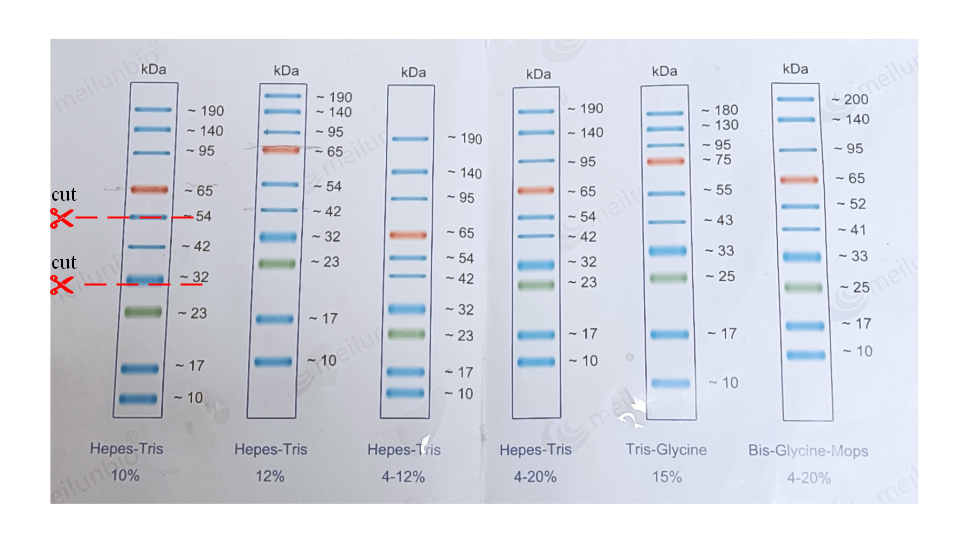

Supplement: S1 File — (ZIP) [file pone.0304185.s001.zip › Single Image-TIF-PACE/Fig5/protein molecular weight.tif]
